# *pi*RNA and Transposon Dynamics in *Drosophila*: A Female Story

**DOI:** 10.1093/gbe/evaa094

**Published:** 2020-05-12

**Authors:** Bastien Saint-Leandre, Pierre Capy, Aurelie Hua-Van, Jonathan Filée

**Affiliations:** Laboratoire Evolution, Génomes, Comportement, Ecologie CNRS, Université Paris-Sud, IRD, Université Paris-Saclay, Gif-sur-Yvette, France

**Keywords:** transposable elements, germline, *pi*RNA, *Drosophila melanogaster*

## Abstract

The germlines of metazoans contain transposable elements (TEs) causing genetic instability and affecting fitness. To protect the germline from TE activity, gonads of metazoans produce TE-derived *PIWI-interacting* RNAs (*pi*RNAs) that silence TE expression. In *Drosophila*, our understanding of *pi*RNA biogenesis is mainly based on studies of the *Drosophila melanogaster* female germline. However, it is not known whether *pi*RNA functions are also important in the male germline or whether and how *pi*RNAs are affected by the global genomic context. To address these questions, we compared genome sequences, transcriptomes, and small RNA libraries extracted from entire testes and ovaries of two sister species: *D. melanogaster* and *Drosophila simulans*. We found that most TE-derived *pi*RNAs were produced in ovaries and that *pi*RNA pathway genes were strongly overexpressed in ovaries compared with testes, indicating that the silencing of TEs by the *pi*RNA pathway mainly took place in the female germline. To study the relationship between host *pi*RNAs and TE landscape, we analyzed TE genomic features and how they correlate with *pi*RNA production in the two species. In *D. melanogaster*, we found that TE-derived *pi*RNAs target recently active TEs. In contrast, although *Drosophila simulans* TEs do not display any features of recent activity, the host still intensively produced silencing *pi*RNAs targeting old TE relics. Together, our results show that the *pi*RNA silencing response mainly takes place in *Drosophila* ovaries and indicate that the host *pi*RNA response is implemented following a burst of TE activity and could persist long after the extinction of active TE families.

## Introduction

In sexually reproducing organisms, germline cells transmit genetic information from generation to generation. The maintenance of genome integrity in these cells is crucial in ensuring the progeny an optimal fitness. Transposable elements (TEs) are selfish genetic elements that have the ability to insert at any genomic location, thus constituting an important source of genetic variability and instability within the germline. In rare cases, the host can take advantage of beneficial TE insertions to establish new genetic functions (Jangam et al. 2017). However, evolutionary trajectories of TEs also rely on negative selective pressures acting against deleterious insertions (Petrov et al. 2003; Le Rouzic and Deceliere 2005; Dolgin and Charlesworth 2008). Indeed, the germline deploys important genetic and epigenetic resources to silence TEs and limit their harmful consequences on host genomes.

Conserved across metazoans, the *PIWI-interacting* RNA (*pi*RNA) pathway is a germline-specific mechanism that plays a predominant role in restricting TE propagation (Lau et al. 2006; Houwing et al. 2007; Kawaoka et al. 2009; Robine et al. 2009). This small RNA-based mechanism involves members of the PIWI family proteins that can bind *pi*RNAs (23–29 nt) and act as transcriptional and posttranscriptional silencers of the TE expression (Brennecke et al. 2007; Czech et al. 2018). Over the two last decades, considerable efforts have been carried out to understand the molecular basis of the *pi*RNA pathway. In *Drosophila melanogaster*, it has been shown that a discrete number of genomic loci, called the *pi*RNA clusters, are dedicated to the production of *pi*RNAs. From these *loci*, which represent <3% of the total genome, hundreds of thousands of different *pi*RNAs are produced and most of them derive from TEs themselves (up to 90% for some clusters, Brennecke et al. 2007). RNA precursors are produced from *pi*RNA clusters and are processed into *pi*RNAs serving as guides to target TE *m*RNAs. Proteins of the PIWI family load *pi*RNAs to mediate both the recognition of complementary TE-derived transcripts and their slicing into small RNAs. Depending on the nature of the *pi*RNA clusters and the PIWI-interacting proteins, populations of *pi*RNAs can eventually feed a secondary amplification process called the *ping-pong* amplification loop. This process leads to massive production of *pi*RNAs against a specific subset of active TE families (Brennecke et al. 2007; Gunawardane et al. 2007; Mohn et al. 2014).

Although models of *pi*RNA biogenesis have been extensively studied from *D. melanogaster* ovaries, they remain poorly studied in the male germline. However, notable differences have been already observed between the *pi*RNA pathway functions in male and female germline. For instance, the PIWI family proteins *Argonaute3* (*Ago3*) and *Aubergine* (*Aub*), known to be essential for the *ping-pong* amplification cycle, display contrasting patterns of expression and cellular localization between the two germlines. On one hand, although *Ago3* protein expression is observed at almost all developmental stages in female germline cells, its expression in testes is restricted to very early stages of spermatogenesis (up to the first four mitotic divisions, Nagao et al. 2010). On the other hand, *Aub* proteins are predominantly associated with TE-derived *pi*RNAs in ovaries, whereas in testes <7% of the *pi*RNAs associated with *Aub* are derived from TEs (Nagao et al. 2010). Indeed, in testes, *Aub* are mainly associated with *pi*RNAs derived from specific Y and X chromosome repeats, but not with transposons (Nishida et al. 2007). Moreover, population analyses in *Drosophila simulans* revealed a general transcriptional bias of both *ago3* and *aub* gene expressions in ovaries compared with testes (Saint-Leandre et al. 2017).

Regardless of lack of understanding in testes, *pi*RNA pathway functions are assumed to serve as the main genome-defense mechanism against new invading TE families. Indeed, the *pi*RNA pathway is regularly compared to an immune system, due to its ability to promptly identify and stop the proliferation of new invading TEs. A number of studies relative to the *P* DNA transposon and the *I* non-long terminal repeat (LTR) retrotransposon have demonstrated that the acquisition of new TE lineages in natural populations is followed by the de novo production of their corresponding *pi*RNAs (Brennecke et al. 2008; Chambeyron et al. 2008; Khurana et al. 2011; Grentzinger et al. 2012). Comparative studies of *D. melanogaster* and its sister species *D. simulans* have shown that the expression of TE-derived *pi*RNAs between populations displays low levels of variation (Akkouche et al. 2013; Song et al. 2014). However, recent populational studies suggest that an increase of *pi*RNA gene expression levels could facilitate TE silencing in *D. simulans* (Lerat et al. 2017; Saint-Leandre et al. 2017). At the genome level, most of the *pi*RNA production likely depends on the presence of TE families that reach a high copy number in the genome, particularly those accumulating within *pi*RNA clusters (Kelleher and Barbash 2013). These observations raise interesting questions regarding the exact relationship between TE activity and their regulation by the *pi*RNA pathway in these two sibling species. Notably, can TE history recapitulate the evolution of lineage-specific *pi*RNA repertoires?

*Drosophila melanogaster* and *D. simulans* are two closely related species that diverged 3–5 Ma (Hey and Kliman 1993; Kelleher and Barbash 2013). Genomic comparison between the two sibling species has revealed that the TE content and landscape are dramatically different (Vieira et al. 1999; Lerat et al. 2011; Kofler, Hill, et al. 2015; Kofler, Nolte, et al. 2015). A range of evidence suggests that the *D. melanogaster* genome has undergone recent transpositional bursts of many TE families (Bowen and McDonald 2001; Bergman and Bensasson 2007; Kofler, Hill, et al. 2015; Kofler, Nolte, et al. 2015). Furthermore, the sequence similarity of the TEs from divergent lineages suggests that the *D. melanogaster* genome has been repeatedly invaded by novel TE families (Sanchez-Gracia et al. 2005; Bartolome et al. 2009; Gilbert et al. 2010). Consistent with the recent activation of many TE families, the genome of *D. melanogaster* contains a large number of full-length copies (Lerat et al. 2011). By contrast, the *D. simulans* genome displays a large number of old and degraded copies indicating that TEs have lost most of their activity (Lerat et al. 2011). Thus, these two sister species represent good models to study the impact of TE evolution and the genome-defense response mediated by the *pi*RNA regulatory machinery.

In the present work, we first reannotated TEs of *D. melanogaster* and *D. simulans* genomes and confirmed that both species display very different TE histories in terms of amplification time and extent. We compared transcriptional levels of TEs in both male and female gonads from several populations of *D. simulans* and *D. melanogaster.* Both species display severe patterns of sex-biased TE transcription in gonads. Comparison of *pi*RNA deep-sequencing libraries showed that ovaries intensely produce *pi*RNAs derived from TEs, whereas TE-specific *pi*RNAs were barely present in testes. We found that variation in the TE content (TE age and structure) between the two species has strongly impacted the populations of *pi*RNAs expressed in the ovaries. Furthermore, we noticed variation in the PIWI pathway (ping-pong efficiency and expression of PIWI effectors) that could also reflect different TE invasion histories. Indeed, *D. melanogaster* ovarian *pi*RNAs preferentially match to TE families overexpressed in testes showing signatures of relatively recent transposition bursts. Although *D. simulans* present signatures of lower TE activity and *pi*RNAs derive from old (fragmented and inactivated) copies, the ping-pong silencing has been efficiently maintained. We propose an evolutionary dynamics model that includes 1) after a new TE invasion, the setup of a progressive implementation of the *pi*RNA machinery in order to moderate and ultimately control TE expansion; 2) after efficient silencing, a long-term persistence of the *pi*RNA production against extinct TE lineages that may help protecting the host from future reinvasions. The differences observed in *D. melanogaster* and *D. simulans* suggest that they may be at different steps of this process.

## Results

### *Drosophila melanogaster* TEs Are Younger and More Abundant to Those Found in *D. simulans*

**Fig. 1.**
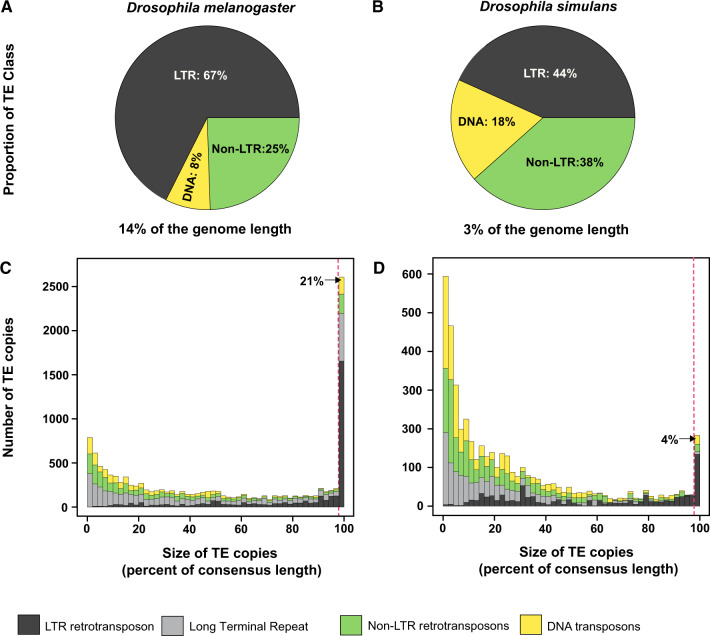
*Drosophila melanogaster* and *Drosophila simulans* share divergent TE histories. Pie charts show the proportion of the different types of TEs in the *D. melanogaster iso1* (dmel r6) (*A*) and *D. simulans w501* (dsim r2) (*B*) genomes. TEs represent 14% of the *D. melanogaster* genome length (based on nonredundant annotations of supplementary table S2, Supplementary Material online) and 3% of *D. simulans* genome length (based on nonredundant annotations of supplementary table S2, Supplementary Material online). Histograms show the size distribution of TE insertions in *D. melanogaster* (*C*) and *D. simulans* (*D*). The *y* axis displays the number of copies found in the reference genome, according to their size. Size of TE insertions was normalized by the length of their respective consensus sequence (*x* axis). Red-dotted vertical lines delimit the full-length elements (≥98% of consensus size) with percentages given. The same color code is used for TE class. We distinguished internal portion of LTR retrotransposons (black) from their LTR (dark gray).

Using a library of *Drosophila* consensus elements derived from Repbase, we first analyzed the relative TE proportions in *D. melanogaster* and *D. simulans*. All main types of TEs are found in both genomes in slightly different proportions (fig. 1*A* and *B*). In *D. melanogaster* and *D. simulans*, LTR retrotransposons constitute the main class of elements (respectively 67% and 44% of the total TE fraction), followed by non-LTR retroelements (25% and 38%, respectively) and DNA transposons (8% and 18%, respectively).

Although both genomes display relative similarities in terms of TE diversity, the total fraction of all repeated sequences (including TEs, satellites, and simple repeats) is quite different between the two species. It represents 15% of the *D. melanogaster* genome (14% of TEs and 1% of other repeated sequences) and 4% of the *D. simulans* genome (3% of TEs and 1% of other repeated sequences). Moreover, genome size differences corroborate levels of TE degradation between the sibling species (fig. 1*C* and *D*). Out of 12,803 TE insertions in *D. melanogaster*, 21% of copies are full length, whereas only 4% of TEs are full length in *D. simulans* (out of 4,583 insertions). These observations are consistent with other comparative studies showing that *D. melanogaster* display abundant full-length TE copies rather than highly degraded copies as in its sibling species *D. simulans* (Lerat et al. 2011).

The genome assembly quality could partially account for this important difference in TE load. The heterochromatic regions of the *D. melanogaster* genome are more contiguous and chromosome arms usually span several additional megabases compared with the *D. simulans* genome. Although the TE excess of the *D. melanogaster* genome (25 Mb) seems a sufficient factor to explain the genome size difference observed with *D. simulans* (175 and 150 Mb, respectively), we compared genomic TE contents on alignable portion of both genomes (i.e., removing deep heterochromatin regions of the *D. melanogaster* assembly). This analysis shows the same qualitative differences between the two species (supplementary fig. S1, Supplementary Material online), notably, a higher genomic TE fraction, higher copy number, and a 5-fold excess of full-length insertions in *D. melanogaster* compared with *D. simulans*.

In summary, we found that the *D. melanogaster* genome TE load is considerably higher compared with that of *D. simulans*, and the copies are less degraded. As suggested before (Lerat et al. 2011), this difference could be explained by different evolutionary histories: a more recent or a continuous TE invasion in *D. melanogaster*, and an older one in *D. simulans*. Such differences in terms of TE histories might have serious consequences on TE transcription levels in the germline.

### TEs Display Strong Sex-Biased Patterns of Expression

**Fig. 2.**
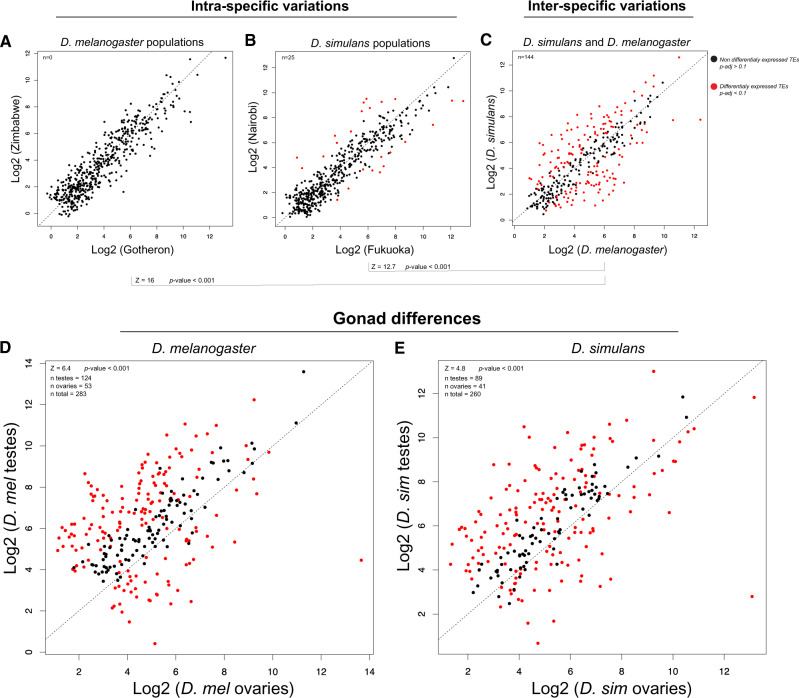
*Drosophila melanogaster* and *Drosophila simulans* TEs display strong sex-biased patterns of expression. Scatterplots (*A*–*E*) showing the transposon expression between species and populations of *D. melanogaster* and *D. simulans*. RNAseq was performed on ovaries and testes and each point shows normalized (DESeq2) values for a transposon family according to conditions. Diagonals represent *x* = *y*. Points in red show TE families significantly differentially expressed (DESeq2, *P* adj < 0.1, FDR = 0.1). (*A*) Relative TE expression between Zimbabwe (*y* axis) and Gotheron (France) populations (*x* axis) of *D. melanogaster*. (*B*) Relative TE expression between Nairobi (*y* axis) and Fukuoka populations (*x* axis) of *D. simulans*. (*C*) Relative TE expression between pooled populations of *D. simulans* (*y* axis) and *D*. *melanogaster* (*x* axis). (*D*) Relative TE expression between testes (*y* axis) and ovaries (*x* axis) of *D. melanogaster* populations. (*E*) Relative TE expression between testes (*y* axis) and ovaries (*x* axis) of *D. simulans* populations. *P* values for differences were obtained by *Z*-statistics.

To understand the relationship between the TE load and their activity in the germline, we sequenced testis and ovary transcriptomes for two populations of *D. melanogaster* (Gotheron and Zimbabwe) and two populations of *D. simulans* (Fukuoka and Nairobi). We mapped these population transcriptomes on the Repbase *Drosophila* TE data set and first computed intraspecific variation of TE transcription. Between populations of the same species, we detected only a limited number of differentially expressed families (fig. 2*A* and *B*). By contrast, comparing TEs between species revealed that 62% of the TE families present in both species (*n* = 232) were differentially expressed (fig. 2*C*). A principal component analysis (PCA) on all data revealed two main axes explaining 61% of the total variance between transcriptomes (supplementary fig. S2*A*, Supplementary Material online). *Drosophila melanogaster* and *D. simulans* are clearly distinguished on the first PCA axis (38%), whereas the second PCA axis (23%) split transcriptomes according to the gonad type (testes vs. ovaries). Differences between sex are confirmed by PCAs performed on each species individually (supplementary fig. S2*B* and *C*, Supplementary Material online). In both cases, the first PCA axis (>55%) is strongly correlated to TE transcription changes between the two germinal lines, whereas differences according to the population captured <20% of the variance on the second axis. Indeed, sex-biased TEs (TEs differentially expressed between male and female germlines) represent 63% of the TE consensus in *D. melanogaster* and 50% in *D. simulans* (fig. 2*D* and *E*). In both species, this sex-biased pattern of expression is mainly due to a global higher expression of TEs in testes. TEs more expressed in testes represent 70% of the sex-biased TEs in *D. melanogaster* and 68% in *D. simulans*. Finally, we observed in both species (supplementary fig. S3, Supplementary Material online) that sex-biased TEs are similarly distributed among the main super-families of TE (i.e., LTR, non-LTR, or DNA transposons).

In summary, two major factors seem to influence TE expression: on one hand, interspecific variation (*D. melanogaster* vs. *D. simulans*) that is much higher than within species, and in the other hand gonad-specific variation (testes vs. ovaries). We observed a global higher expression of most TE families in testes compared with ovaries for both populations of *D. melanogaster* and *D. simulans*. We also observed some variations between populations within each species, as previously reported (Lerat et al. 2017). Yet, intraspecific variations remain much lower than those observed both between the two species and between sex (supplementary table S1, Supplementary Material online).

### *pi*RNA-Mediated Silencing of TE Is Predominant in Ovaries but Weak in Testes

Could the global TE overexpression in testes underlie major sexual differences of the *pi*RNA regulatory pathway between gonads? To evaluate this hypothesis, we first compared levels of TE-derived *pi*RNAs across ovaries of different laboratory strains and populations for which small RNA sequences were publicly available (see supplementary table S1, Supplementary Material online). We observed only slight variation (supplementary fig. S4*A*–*C*, Supplementary Material online). Indeed, <4% of TEs show a *pi*RNA expression change higher than 2-fold. This result agrees with previous independent studies showing that pools of ovarian *pi*RNAs were stable across strains and populations (Akkouche et al. 2013; Song et al. 2014).

**Fig. 3.**
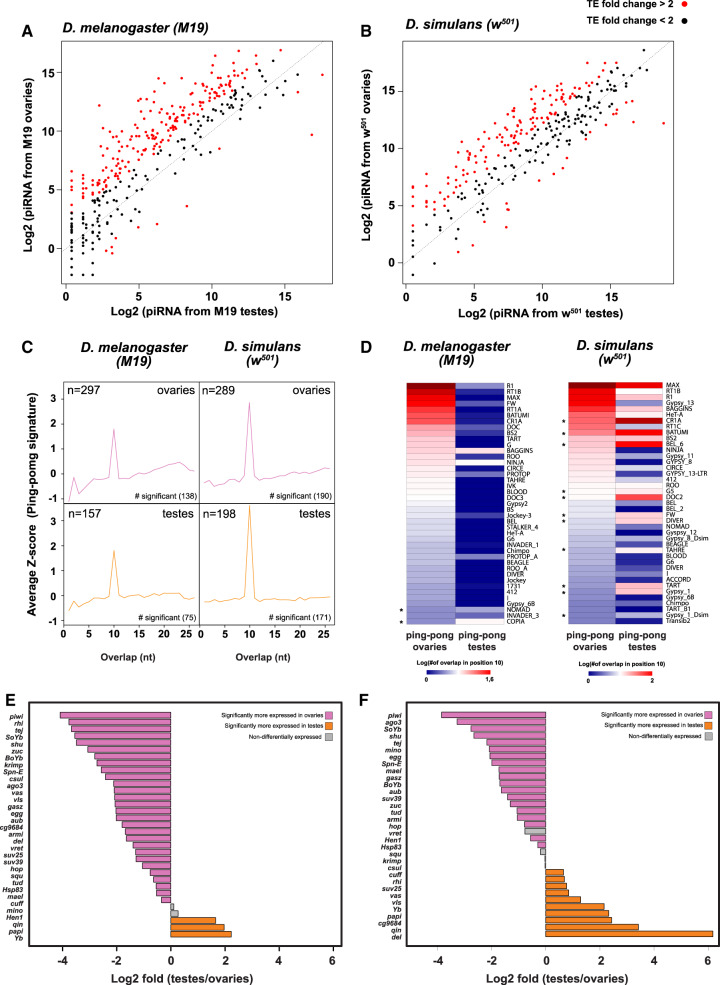
*pi*RNA-mediated silencing of TE is predominant in ovaries, weak in testes. (*A*, *B*) Scatterplots showing the *pi*RNA expression (number of *pi*RNA normalized by total number of miRNA) per TE family between ovaries and testes of *Drosophila melanogaster* (*A*) and *Drosophila simulans* (*B*). Diagonals represent *x* = *y*. Points in red show TEs displaying a fold change expression >2. (*A*) Relative *pi*RNA expression from ovaries of the *D. melanogaster* w^1118^-derived strain M19 ovaries(*y* axis) and M19 testes (*x* axis). (*B*) Relative *pi*RNA expression from ovaries of the *D. simulans* strain *w^501^* (*y* axis) and *w^501^* testes (*x* axis). (*C*, *D*) Plots showing probability of overlapping *pi*RNAs and the length of the overlap according to their starting position on 3′ *pi*RNAs in *D. melanogaster* (left) and *D. simulans* (right). Pink lines show average *z*-score in ovaries, whereas orange lines show average *z*-score in testes. Total number of TE families presenting overlapping *pi*RNAs and number of TE families presenting significant overlap (*z*-score > 1.96; *P* < 0.05) are indicated. (*D*) Heatmaps comparing *ping-pong* signatures in testes and ovaries for a set of TEs shared between *D. melanogaster* (left) and *D. simulans* (right). The *ping-pong* signature is expressed as the number of overlapping pairs (first 10 nt) normalized by number of *pi*RNAs. Black asterisks highlight TEs with stronger *ping-pong* signatures in testes. Bar graphs (*E*, *F*) shows relative expressions of *pi*RNA pathway genes between ovaries and testes of *D. melanogaster* (*E*) *and D. simulans* (*F*). Pink bars show genes more expressed in ovaries. Orange bars show genes more expressed in testes. Genes that are not significantly differentially expressed are shown in gray.

For sex comparisons, we used data set of laboratory strains, produced in this study or publicly available (M19, a strain derived from w^1118^ for *D. melanogaster* and w^501^ for *D. simulans*). Between male and female gonads, not only *pi*RNAs but also other small RNA species greatly differ (supplementary fig. S5, Supplementary Material online). Indeed, *pi*RNAs and miRNAs in testes represent a small fraction of the total small RNA pool compared with ovaries (supplementary table S1, Supplementary Material online). These ostensible differences may reflect the very distinct biological functions carried out in male and female germlines and direct comparisons may be challenging. To avoid eventual bias linked to products of mRNA degradation, we normalized *pi*RNAs by the total number of miRNAs. In both species, after normalization, the observed *pi*RNA drop in testes still persists when compared with ovarian *pi*RNAs (supplementary fig. S5, Supplementary Material online). In *D. melanogaster* ovaries, 52% of TE families display a >2-fold *pi*RNA enrichment compared with testes (fig. 3*A*). This pattern is conserved in *D. simulans* where 47% of TEs show such a biased expression in ovaries (fig. 3*B*). Indeed, significant changes correspond almost exclusively to a higher production in ovaries. These observations were conserved as well, when we mapped *pi*RNAs with other mismatch thresholds (supplementary fig. S6*A* and *B*, Supplementary Material online).

The *ping-pong* process is a secondary amplification of *pi*RNAs generated from the slicing of mRNA precursors. Slicing of precursors generates secondary sense *pi*RNAs that typically overlap by 10 nt with the complementary antisense guiding *pi*RNAs (Brennecke et al. 2007). An excess of 10-nt overlap observed between sense and antisense *pi*RNAs is then the signature of a *ping-pong* mechanism. We computed the length of overlap in our data set (Antoniewski 2014) and detected global significant *ping-pong* signatures in both gonads of both species. However, comparing the overlap signals for a set of TE consensus shared by the two sibling species, we noticed that *ping-pong* signals were predominantly higher in ovaries than in testes (fig. 3*D*). TE overlap signatures in *D. simulans* testes are stronger compared with *D. melanogaster* testes (fig. 3*C*), suggesting that the *ping-pong* mechanism is more efficient in *D. simulans* testes. Consistent with this observation, TE families with higher *ping-pong* signal in testes than in ovaries (fig. 3*D*) are more important in *D. simulans* (11 out of 40) compared with *D. melanogaster* (2 out of 40).

Using our RNAseq data, we further compared male and female gonad patterns of expression (fig. 3*E* and *F*) for a set of genes known to be essential for *pi*RNA silencing (Handler et al. 2013). In *D. melanogaster*, 85% of the *pi*RNA regulatory genes are more expressed in ovaries. In *D. simulans*, a similar trend is observed with 58% of *pi*RNA genes showing enriched expression in ovaries. The number *pi*RNA genes with higher expression in testes is greater in *D. simulans* (30%) compared with *D. melanogaster* (9%). Interestingly, this pattern is consistent with a more efficient *ping-pong* amplification loop in *D. simulans* testes.

Altogether, these data suggest that the TE silencing via *pi*RNAs presents a female-biased activity: 1) expression of genes involved in the *pi*RNA pathway is higher in the female germline, 2) ovaries produce remarkably larger amounts of *pi*RNAs derived from TEs than testes do, and 3) TE families have generally higher *ping-pong* signatures in ovaries than in testes. This pattern could be a major contributor of the TE overexpression pattern in testes evidenced in this study. Nevertheless, based on these observations, it remains difficult to propose a comprehensive view of the relationship between the germline *pi*RNA repertoire, the TE sex-specific patterns of expression, and the TE dynamics within and between genomes. To this end, we performed qualitative and quantitative analyses of *pi*RNA variation to understand the genomic features of TEs preferentially targeted.

### TE Histories Have Shaped the Dynamics of *pi*RNA Biogenesis

**Fig. 4.**
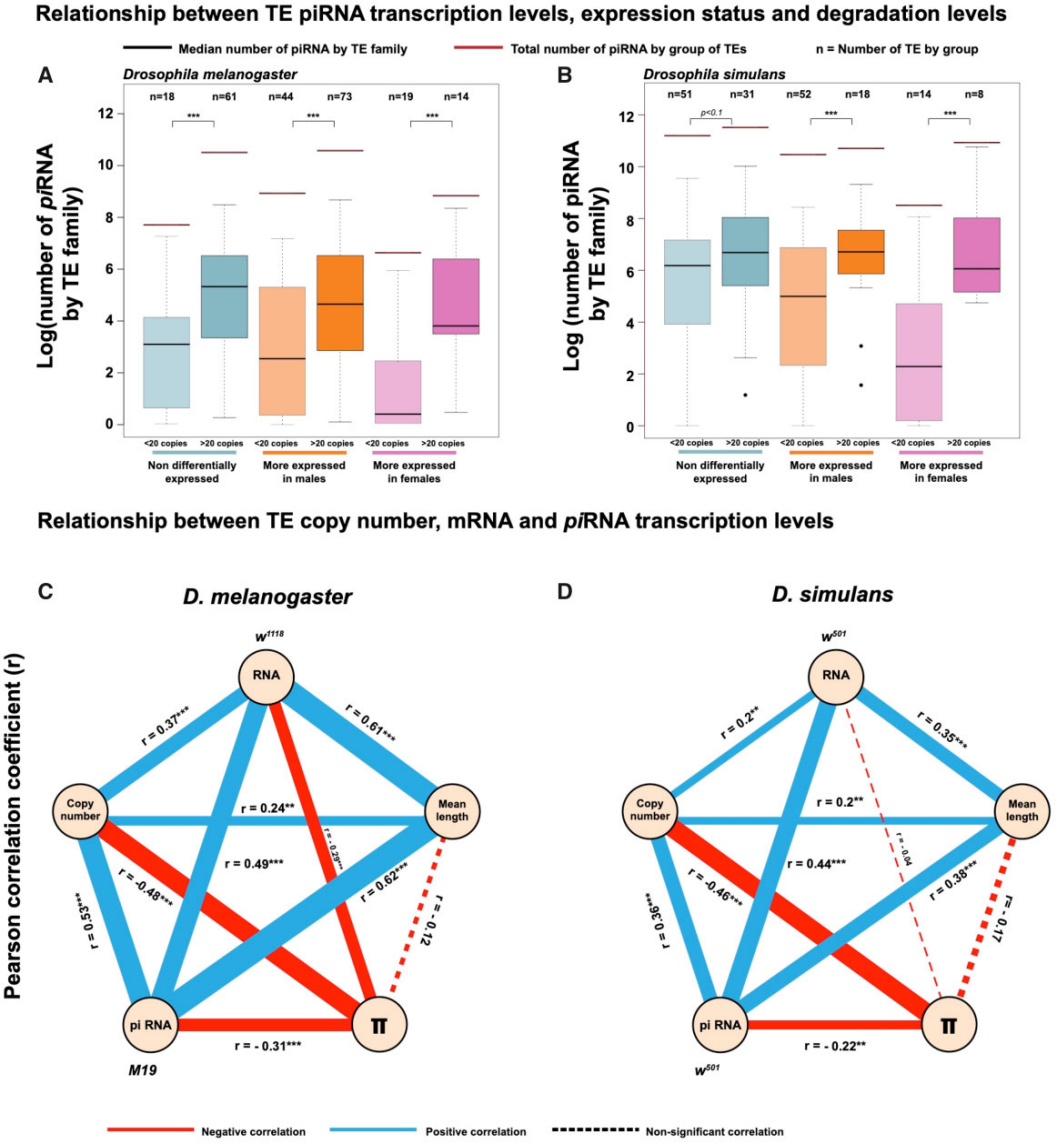
Relationship between TE piRNA transcription levels and genomic features of TEs. (*A*, *B*) Boxplots show the distribution of the normalized number of ovarian *pi*RNA per TE family in *Drosophila melanogaster* (*A*) and *Drosophila simulans* (*B*). Clear colors represent TE families present in <20 copies and dark colors represent TE families displaying more than 20 copies in the genome. Blue shows nondifferentially expressed TE families, pink is for TE families more expressed in ovaries, and orange for TE families more expressed in testes. Stars indicate *P* value of Wilcoxon statistics. (*C*, *D*) Pearson’ correlation coefficients between *pi*RNA transcription levels and TE genomic traits (i.e., mRNA transcription levels, number of copies, median length of the TE families, and the nucleotide diversity index *π*) in *D. melanogaster* (*C*) and *D. simulans* (*D*). Dashed lines represent nonsignificant correlations and solid lines significant correlations. Thickness of the lines is proportional to their respective Pearson’ coefficients *r*. Blue lines stand for positive correlations, whereas red for negative correlations. The significant *P* values are indicated for each solid line.

The *pi*RNA-mediated silencing of TEs might have been primarily co-opted to slow down transposition rates of the most active families. However, an efficient silencing could eventually suppress the activity of a targeted lineage. In this context, an intense *pi*RNA response could reflect either a recent transposition burst or an abundant TE family that stop transposing and start to go extinct. Here, we analyzed the relationship between amplification levels of TE families and the strength of their specific *pi*RNA responses (fig. 4*A* and *B*) using ovarian *pi*RNA data sets presented in the previous section (fig. 3*A* and *B*). We first used copy number (more or <20 copies in the reference genome) as an arbitrary criterion to distinguish between highly and poorly amplified TE families and grouped TEs according to their expression status (more expressed in testes, in ovaries, or nondifferentially expressed). We detected a clear relationship between *pi*RNA levels and TE abundance in the genome. In both species, the average number of *pi*RNA *per* TE was significantly higher for the most amplified TE families (>20 copies) compared with less abundant families (<20 copies), whatever their status concerning differential expression between gonads. In addition, we observed the same pattern for *pi*RNA production in testes (supplementary fig. S7, Supplementary Material online), suggesting that *pi*RNAs of both germlines are mainly responding to the most abundant TE families. These results suggest that *pi*RNAs primarily respond to the most abundant TE lineages in the genome although copy age and degradation are strikingly different between the two focal species.

To provide additional supports to this observation, we performed a more complete analysis of the relationship between the genomic characteristics of each TE family and the strength of the corresponding *pi*RNA defense response (fig. 4*C* and *D*). PIWI proteins loaded with *pi*RNAs target TE mRNAs and degrade TE transcript through their slicing activity. During the *ping-pong* cycle, PIWI proteins directly use TE mRNAs as substrate to generate novel *pi*RNAs indicating that TE mRNA levels may have critical impacts on *pi*RNA biogenesis output. Consistent with this phenomenon, both species display a strong positive relationship between mRNA and *pi*RNA levels (*r* = 0.49*** for *D. melanogaster* and *r* = 0. 44*** for *D. simulans*). Levels of TE transcription are mainly explained by an increase in TE copy number (*r* = 0.37*** for *D*. *melanogaster* and *r* = 0.2** for *D*. *simulans*) and TE length (*r* = 0.61*** for *D. melanogaster* and *r* = 0.35*** for *D. simulans*). The nucleotide diversity index (*π*) is related to the conservation level between copies of a given TE family (a low *π* corresponds to highly similar copies indicating a recent expansion). We observed a relationship between transcription levels and *π* in *D. melanogaster* (*r* = −0.29***) but not in *D. simulans* (*r* = −0.04 for *D*. *simulans*).

However, in both species, the *pi*RNA production is significantly associated with these four variables that altogether describe the intensity of TE family’s activity. Levels of *pi*RNAs dramatically increase for TE families displaying high copy number, high length, and low *π* (*r* = 0.53***, 0.62***, and −0.31***, respectively, for *D. melanogaster* and *r* = 0.36***, 0.38***, −0.22**, respectively, for *D. simulans*), suggesting that production of *pi*RNAs preferentially target expanding, or expanded but not too old, TE families. These trends are conserved regardless of the data set used (RNAseq, small RNAseq, or genomes, see supplementary fig. S7 and table S3, Supplementary Material online). However, relationships between *pi*RNA and recent activity markers are globally weaker in *D. simulans* (old TE invasions) compared with *D. melanogaster* (more recent TE invasions), which support a preferential link between *pi*RNA production and recent TE expansion (fig. 4*C* and *D*).

In summary, differences between the sibling species appear to be the result of different tempo and activities of TE invasion: a recent invasion in *D. melanogaster* where TEs spread actively and an ancient invasion in *D. simulans* where TEs slowly go extinct. These results suggest that the host *pi*RNA-mediated defense was activated first to slow down the invasion of the most active TE lineages and later to maintain a long-term protection against former successful TEs. If this assertion is true, we should observe 1) an accumulation of the most active TE families within *pi*RNA clusters and 2) their persistence within *pi*RNA clusters when TE families get old and lose their activity.

### Recent Bursts of Transposition Enhance the Ovarian Specificity of *pi*RNA Clusters

**Fig. 5.**
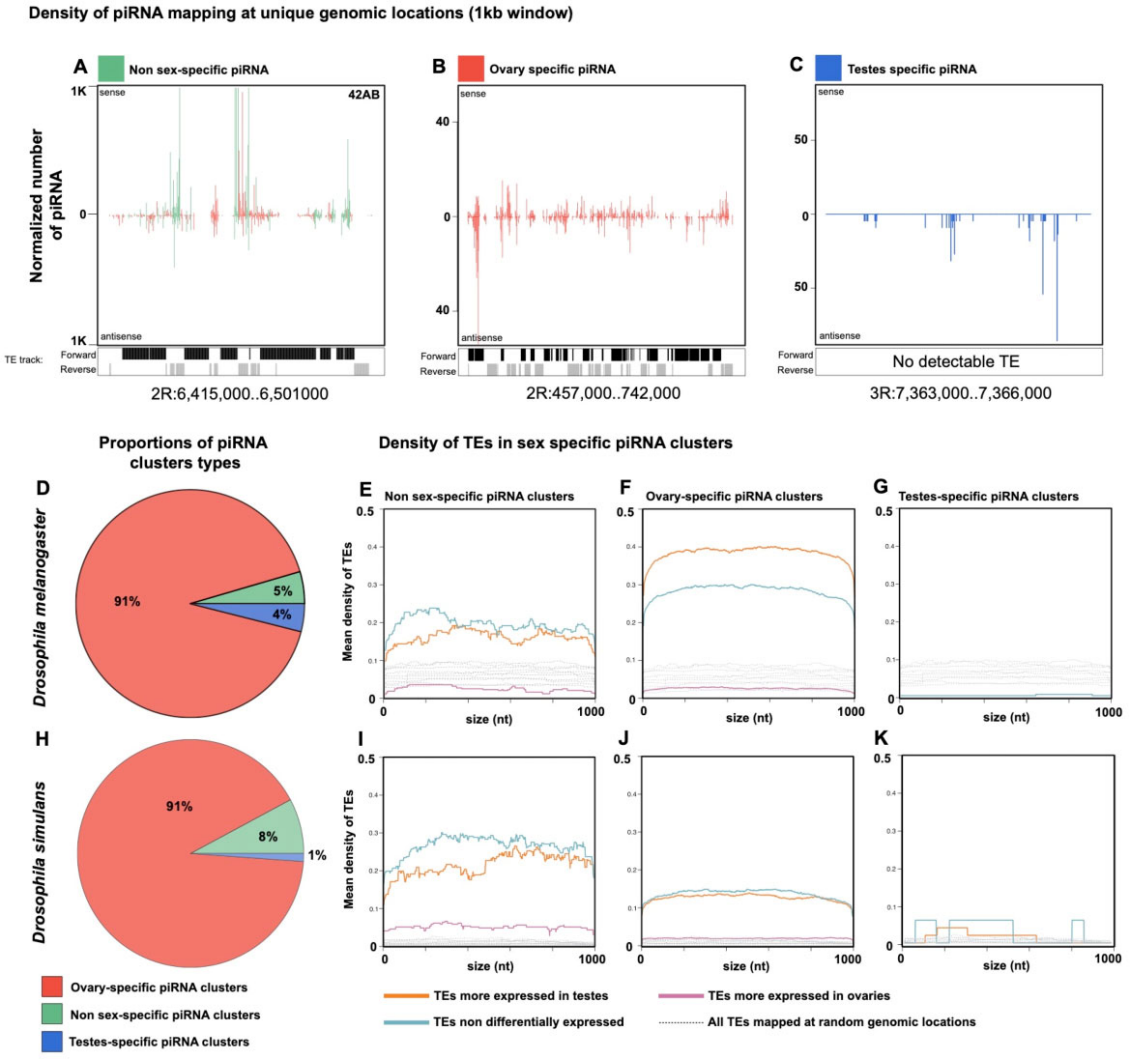
Sex-biased evolution of *pi*RNA clusters is shaped by the tempo of TE activity. The genomic maps (*A*–*C*) show the number of uniquely mapping *pi*RNAs along the regions of chromosome 2R (*A*, *B*) and 3R (*C*) of *Drosophila melanogaster* species (*x* axis). Colored bars show the number of *pi*RNAs according to their expression pattern: green bars display *pi*RNAs expressed in both testes and ovaries, red bars *pi*RNAs exclusively expressed in ovaries, and blue bars *pi*RNAs exclusively expressed in testes. All small RNAs display a size comprised between 23 and 30 nucleotides. Composition in TEs is indicated underneath: black shows TEs inserted in forward orientation and gray TEs inserted in reverse orientation relative to the genome. (*D*, *H*) Pie charts showing the relative proportion of *pi*RNA clusters across the whole genome. (*E*–*G*, *I*–*K*) The average TE density (*y* axis) along the 1-kb window of *pi*RNA clusters screened all along the genome (*x* axis). The TE density was analyzed according to the sex expression pattern of TE families. Pink lines represent TEs more expressed in females, blue lines stand for TEs nondifferentially expressed, and orange lines for TEs more expressed in testes. The *pi*RNA clusters were analyzed according to their germline expression. (*E*, *I*) Stand for *pi*RNA clusters expressed in both testes and ovaries, (*F*, *J*) for *pi*RNA clusters exclusively expressed in ovaries, and (*G*, *K*) for *pi*RNA clusters exclusively expressed in testes.

To investigate the dynamics of TEs within *pi*RNA-producing loci and the consequences on sex-biased expression, we compared the *pi*RNA clusters density of TEs according to their expression pattern (overexpressed in testes, overexpressed in ovaries, and nondifferentially expressed). In both species, we localized *pi*RNA clusters and compared their genomic distribution in testes and ovaries (fig. 5*A*–*C*). The cluster 42AB, known as a *pi*RNA “master locus” in *D. melanogaster* (Brennecke et al. 2007), is transcriptionally active in both testes and ovaries (fig. 5*A*). Along this genome, however, we could also identify other clusters that are expressed only in ovaries (e.g., pericentric *pi*RNA cluster of the chromosome 2R, fig. 5*B*), and clusters that are transcriptionally active only in testes (fig. 5*C*). We performed a global screen of the genome for *pi*RNA-producing loci in a 1-kb window and revealed that most of the *pi*RNA clusters are active in ovaries (fig. 5*D* and *H*). In *D. melanogaster*, 4% of *pi*RNA loci are testes specific, 5% were found expressed in both germline, and 91% were only expressed in ovaries. In *D. simulans*, female-specific *pi*RNA clusters represent as well 91% of all *pi*RNA clusters. These data clearly reinforce our previous observations that the female germline is the main tissue involved in TE silencing and also explain the global tendency to produce less *pi*RNA in testes.

We further characterized TE density along *pi*RNA clusters according to their pattern of expression (fig. 5*E*–*G* and *I*–*K*). In *D. melanogaster*, we observed that TEs more expressed in testes display the highest density within female-specific *pi*RNA clusters. These TEs constitute almost 40% of ovary-specific *pi*RNA clusters. Nondifferentially expressed TEs are predominant (∼20%) in *pi*RNA clusters active in both germlines, closely followed by TEs with enriched expression in testes (∼15%). In contrast, TEs with higher expression in ovaries exhibit an extremely low density across all *pi*RNA clusters. Interestingly, in *D. simulans*, we noticed a lower TE density within ovary-specific *pi*RNA clusters (fig. 5*J*) compared with nonspecific ones (fig. 5*I*) and also compared with *D. melanogaster* (fig. 5*F*). This pattern in TE density in *D. simulans* could be a direct consequence of the TE degradation process, suggesting that ovary-specific *pi*RNA clusters are progressively purged from TEs once their invasion has been successfully tackled.

Therefore, it seems that when a species experiences an intense TE expansion (as for *D. melanogaster*), an accumulation of TE fragments occurs in genomic regions dedicated to the ovarian-specific production of silencing *pi*RNAs leading to a contrast of TEs expression in the two germlines. Then, when the TE expansion is under control (as for *D. simulans*), a progressive TE loss in these exclusively female *pi*RNA clusters occurs, which re-equilibrates the pattern of TE expression between testes and ovaries.

## Discussion

### Divergent TE Evolutionary History between *D. melanogaster* and *D. simulans*

Our results show that during 5 Myr of divergence, *D. melanogaster* and *D. simulans* genomes have accumulated very different TE content. This is consistent with several previous studies comparing the two sibling species (Vieira et al. 1999, 2012; Lerat et al. 2011). Recently, a large-scale analysis from natural populations of both species has revealed that most of the new TE insertions are due to the ongoing expansion of 58 TE families (Kofler, Nolte, et al. 2015). In addition, the distribution among populations of these recently invading TEs described high levels of heterogeneity, consistent with some non-annotated TE families in the reference genomes. In our study, we only consider TE families that have been present in the reference genome, excluding low frequency TE lineages that are not yet established. For instance, the ongoing *P* element invasion of *D. simulans* (Kofler, Hill, et al. 2015) is not present in the reference genome. However, our estimations of the TE diversity in the reference genome (fig. 1) and in other assemblies (supplementary table S3, Supplementary Material online) are similar to those determined by pool sequencing analyses (Kofler, Nolte, et al. 2015). Besides the global TE load, the most striking difference observed here is the strong overrepresentation of deleted copies in *D. simulans* compared with *D. melanogaster*. This suggests that the main differences between both species are related to the tempo of TE activity: *D. melanogaster* could be characterized by recent TE invasion or transposition bursts of several TE families, whereas *D. simulans* TE content consists mainly in fragmented and inactive elements probably due to ancient invasions.

Transcriptomic data of gonads show that differences in TE expression between populations are quite limited compared with those detected between male and female germlines. We have therefore concentrated our analyses on TE families differentially expressed in these tissues. Interestingly, we found that levels of TE transcription were related to TE copy number and *pi*RNA levels of expression in the germline. Because the *pi*RNA pathway is crucial in modulating TE activity in the germline, we further analyzed the type of relationships between features of TE activity and the subsequent host-mediated silencing response.

### TE Activities and the *pi*RNA Genome Response

We analyzed TE-derived *pi*RNA profiles in order to clarify the relationship between TE activity and *pi*RNA regulation. We observed that the majority of the TE-derived *pi*RNAs matched to TE families that are highly transcribed. The positive correlation between *pi*RNA and TE mRNA levels likely result from the PIWI protein slicing activity that use TE mRNAs as substrates to generate novel *pi*RNAs during the *ping-pong* amplification cycle. At the genome level, the response against TE invasions is predominantly achieved by multiple insertions within *pi*RNA clusters involved in the secondary *pi*RNAs biogenesis. These observations suggest that a TE family inserted at high density within *pi*RNA clusters, and still producing abundant mRNA transcripts would represent an ideal *pi*RNA target.

Consistent with Kelleher and Barbash (2013) model, we observed that high levels of mRNA and *pi*RNA expression are features associated with TE families displaying higher copy number, suggesting that the host TE silencing response was shaped by successful TE amplification. In addition, we observed a strong relationship between *pi*RNA levels and features of recent TE activity (long length and low diversity between copies) indicating that *pi*RNAs preferentially target relatively recent waves of TE expansion. However, these correlations persist in *D. simulans* where TEs are more degraded and less active compared with *D. melanogaster*. This last observation suggests that among a pool of ancient TEs, the relatively youngest families will still be preferentially targeted by *pi*RNAs. Altogether, our data favor a model in which *pi*RNA production is acquired during TE expansion, as soon as copies are accumulated and fixed in *pi*RNA clusters. Then, the maintenance of an active *pi*RNA production relies on the absolute mRNA levels of a given TE family, its rate of degradation in the genome, and ultimately, on its rate of elimination from *pi*RNA clusters.

Indeed, *pi*RNA clusters are composed of repeated sequences derived from TEs and their fragmented derivatives (Brennecke et al. 2007). Their genomic locations are conserved across *Drosophila* species suggesting that natural selection has favored the maintenance of TE silencing regions producing *pi*RNAs (Brennecke et al. 2007; Malone and Hannon 2009; Castaneda et al. 2011). It has been shown that the pool of TEs within a *pi*RNA cluster can be easily updated by new TE insertions (Malone and Hannon 2009; Khurana et al. 2011), indicating that the TE composition of the *pi*RNA clusters might be directly dependent on the species pool of successfully invading TEs. In addition, models of TE dynamics predict that TE can take advantage of the *pi*RNA silencing machinery to reach fixation within *pi*RNA clusters (Lu and Clark 2010; Kofler 2019).

*pi*RNA clusters are located in highly heterochromatic regions (Brennecke et al. 2007). Then, a new TE insertion inside these regions may confer numerous advantages in a selective context. Such an insertion is not deleterious to the host and may ultimately give the host the ability to silence other TEs due to similarities between TEs. In contrast, euchromatic copies are often associated with deleterious effects and thus frequently removed by purifying selective forces (Gonzalez et al. 2008; Lu and Clark 2010). This scenario is consistent with the positive correlation observed between copy number, *pi*RNA abundance observed in the present work, and the predominance of heterochromatic TE insertions in both species (Junakovic et al. 1998; Bartolome et al. 2002; Kaminker et al. 2002). In this respect, the comparison between *D. melanogaster* and *D. simulans* is of particular interest because they display different tempos of TE activity. Compared with *D. melanogaster*, *D. simulans* presents a dramatic TE loss characterized by a low copy number and a lower TE size (e.g., fig. 1). However, despite lower TE density within *pi*RNA clusters in *D. simulans*, these TE fragments are sufficient enough to maintain a TE *pi*RNA production (figs. 3 and 5). This may be due to the efficiency of the *D. simulans* PIWI pathway. In any case, it seems that once established, the *pi*RNA silencing persists until the complete decay of the ancient families. In the late steps of invasion, although full-length active elements keep on declining or become extinct, copies are still able to persist as small TE relics embedded within the *pi*RNA clusters and act against transcription of euchromatic ones.

### The TE *pi*RNA Regulatory Machinery Is a Female-Specific System

Only very few works have paid attention to TE silencing by the *pi*RNA pathway in testes. First, it was shown that most *pi*RNAs derived from *Stellate* in *D. melanogaster* (supplementary fig. S5 and table S1, Supplementary Material online, and Nishida et al. [2007]). A biochemical approach evidenced that most of the *pi*RNAs derived from TEs are loaded by *Ago3* but not by *Aub*, and that *Ago3* expression is restricted to the first four cellular divisions in *D. melanogaster* (Nagao et al. 2010). These results are consistent with our observations showing that in *D. melanogaster* testes, *pi*RNAs constitute a weak fraction of our small RNA libraries and generally display weaker *ping-pong* signatures (fig. 3*D*). The TE-derived *pi*RNAs observed in testes are likely to be analogous to those described by Nagao et al. (2010) and are thus probably restricted to the *Ago3*-loaded *pi*RNA at the extreme part of the testes. Indeed, TE-derived *pi*RNA populations collapse when testicular germ cells differentiate into spermatocytes (Quénerch’du et al. 2016). Here, we compared small RNA libraries of testis developmentally arrested mutants in early mitotic division (Quénerch’du et al. 2016) to our libraries from entire wild type testes. Only 10% of TEs display significant differences between these two conditions (supplementary fig. S3*D*, Supplementary Material online), suggesting that most of the *pi*RNA production in testes is limited to the very first stages of cell differentiation. Moreover, we mapped testes *pi*RNA clusters in both *D. melanogaster* and *D. simulans* and found that *pi*RNA clusters exclusively active in testes are rare and have a very low TE density. Indeed, testes *pi*RNA clusters containing TEs are the ones also active in females. Altogether, these results suggest that the role of *pi*RNA-mediated TE silencing in testes is relatively limited.

We suggest that this large difference directly contributes to the overall bias of TE expression in testes. In *D. melanogaster*, 119 TE families are overexpressed in testes, whereas they are mainly silenced in ovaries. This trend is more balanced in *D*. *simulans* for which only 70 TE families were testes biased.

Levels of *pi*RNAs and *ping-pong* signatures are higher in *D. simulans* testes than in *D. melanogaster*, indicating that a more efficient *pi*RNA production in testes could reduce this bias.

### Sex-Biased Evolution of *pi*RNA Clusters

More than 90% of *pi*RNA clusters are exclusively expressed in ovaries. In *D. melanogaster*, TEs are recent active lineages, whereas mostly degraded in *D. simulans*. Our results show that TEs cover ∼60% of female-specific *pi*RNA clusters in *D. melanogaster* and ∼20% in *D. simulans*. These observations indicate that female-specific *pi*RNA clusters become saturated in TEs during current invasions and are progressively purged once invasions have stopped. As opposed to female-specific clusters, TE density of nonsex-specific clusters still remains high in *D. simulans*. These results suggest that selection is favoring TE accumulation within female-specific *pi*RNA clusters during pervasive expansion and that selection maintains TEs within *pi*RNA clusters expressed in both sexes once TE activity has been controlled by the host.

The evolutionary arms race between host and TEs also has direct consequences on the evolution rate of *pi*RNA effector proteins (Kidwell and Lisch 2001; Aravin et al. 2007; Siomi et al. 2008; Blumenstiel 2011; Lee and Langley 2012). Independent works support that *pi*RNA proteins belong to the faster evolving component of coding sequences in *Drosophila* genomes and are further subject to recurrent adaptive mutations (Heger and Ponting 2007; Pane et al. 2007; Berry et al. 2009; Obbard et al. 2009; Kolaczkowski et al. 2011; Lee and Langley 2012). In this study, *pi*RNA genes display different patterns of expression between the two sibling species, suggesting that host proteins have adapted to species-specific constraints. Indeed, we observed stronger *ping-pong* signatures in *D. simulans* compared with *D. melanogaster*. In terms of evolutionary strategies, a more efficient *pi*RNA amplification may suppress TE activity more promptly and in the end make the host less permissive to new TE invasions (Lerat et al. 2017; Saint-Leandre et al. 2017). In this context, shaping the efficiency of the *pi*RNA machinery could reflect an adaptation reminiscent of former pervasive transposition. Alternatively, it could reflect an adaptation to compensate the lack of TE material required to generate novel *pi*RNAs when genome TE content is too low.

Despite these species specificities, we found that the majority of essential *pi*RNA genes (Handler et al. 2013) display patterns of overexpression in ovaries. It is usually expected that genes under sex-specific selection display a sex-biased expression (Ellegren and Parsch 2007). Under this assumption, the *pi*RNA regulatory genes are under a strong female selection in both species. However, the female-biased expression of *pi*RNA pathway genes is more balanced in *D. simulans* indicating that female-specific selective pressures were relaxed because TE propagation has been stopped.

Altogether, our results indicate that increased TE activity may enhance female-biased investments in mobilizing genomic defense resources. However, an increase of TE activity in the male germline may constitute an important source of genetic variability and rearrangements that can feed the emergence of new genetic conflicts. In a wide range of species, male germline was described as a crucial tissue driving the evolution of genomes. The male-driven hypothesis was built on the observation that mutation rates in male gametes is always higher compared with female gametes (Hurst and Ellegren 1998; Connallon and Knowles 2005; Connallon and Clark 2010; Parsch and Ellegren 2013). Another concept, named the “out of testes” hypothesis, is based on the observation that the vast majority of newly emerging genes start to be expressed in a testes-specific manner (Paulding et al. 2003; She et al. 2004; Levine et al. 2006; Ponce and Hartl 2006; Heinen et al. 2009; Kaessmann 2010; Light et al. 2014). In this context, TEs were shown to stand as important contributors of the “male-biased” evolution of genomes (Bennetzen 2000; Toll-Riera et al. 2009; Wilson Sayres and Makova 2011; Wissler et al. 2013). In the future, it would be challenging to test to what extant TEs in testes can constitute a force that facilitates genetic rearrangements and thus, to what extant TEs in testes enlarge the selective spectrum required to promote diversifying selection and adaptive innovations.

## Conclusion

**Fig. 6.**
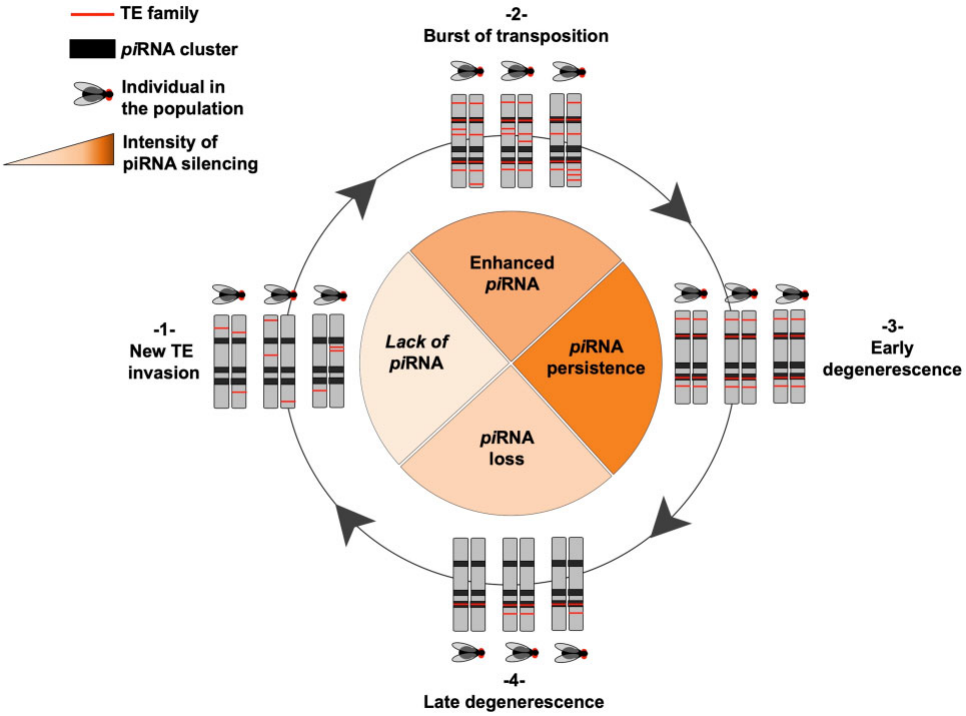
Evolutionary dynamics of a new emerging TE family under control of the *pi*RNA silencing pathway. Step 1 represents a young emerging TE (red line) in a population of diploid genomes (pair of gray bars). The activity of the new element is associated with a strong insertion polymorphism, and thus present at diverse genomic locations that are not fixed in the population. Step 2 corresponds to the establishment of the family within the population of genomes. Following the TE burst of amplification, some TE insertions are now found to be fixed in many genomic loci and some of them into *pi*RNA-producing regions (black line). This is the stage found in *Drosophila melanogaster.* These insertions appear selectively advantageous for the host as they are able to limit the expansion of the TE family. Step 3 corresponds to the long-term establishment of the TE family: Due to the implementation of the *pi*RNA machinery, most of the TE insertions are now fixed and not able to transpose. The *pi*RNA response will persist until step 4 as observed in *Drosophila simulans*. Step 4 corresponds to the very long-term dynamics in which most of the TE insertions are found completely degenerated and fragmented. TEs are progressively removed from the genome and the loss of the fragmented copies inserted in the *pi*RNA cluster lead to the progressive loss of the genomic *pi*RNA response. Once completely lost, the cycle is over and a new reinvasion can occur.

In this work, we found that the tempo and the dynamics of TE invasion are clearly different between two closely related species of *Drosophila*: *D. simulans* have experienced ancient waves of TE invasion, whereas *D. melanogaster* still undergo recent TE bursts. We proposed that trajectories of TE invasion have strongly affected the host defense machinery involved in TE silencing through the production specific pools of *pi*RNAs. Moreover, we found that the “postinvasion” *pi*RNA-mediated response is dramatically enhanced in ovaries compared with testes. Therefore, we proposed a dynamic model describing how the *pi*RNA silencing machinery takes place through the female germline (fig. 6).

In the early stage of the invasion, new TE insertions are characterized by a high insertion polymorphism and fixations at a given locus are rare. As a consequence, these recently expanding TE families are rarely targeted by *pi*RNAs. Alongside the TE amplification process, the targeted *pi*RNA response becomes progressively active as some insertions become fixed into *pi*RNA clusters. This step corresponds to what we observe in *D. melanogaster*. Thereafter, when *pi*RNA posttranscriptional silencing is stably established, the mobilization of active TEs considerably slows down. In the long term, as observed in *D. simulans*, although active copies will progressively degenerate and finally disappear (except few relic copies in *pi*RNA clusters), the persistence of the *pi*RNA response will act as a long-term genomic memory to protect the genomes from future invasions.

## Materials and Methods

### *Drosophila* Stocks

*Drosophila* natural populations were collected from geographically distinct area. We used *D. melanogaster* populations from Zimbabwe (Harare) and Gotheron (France) and *D. simulans* populations from Nairobi (Kenya) and Fukuoka (Japan) for transcriptome analysis. Populations were maintained at 25 °C from the date of capture as well as laboratory strains. We also used the laboratory strains M19 and *w^1118^*of *D. melanogaster*, the strain *w^501^* and natural population from Makindu (Kenya) and Chicharo (Portugal) of *D. simulans* for small RNA sequencing analysis. The list of populations and strains is given in supplementary table S1, Supplementary Material online.

### mRNA Library Preparation and Small RNA Library Preparation

We extracted total RNA from 30 pairs of ovaries and testes in 2–4-day-old adults, according to the manufacturer’s instructions (Macherey-Nagel). PolyA mRNAs were extracted using the “FastTrack MAG Micro mRNA isolation kit” (Life Technologies), fragmented with RNA fragmentation reagents (Ambion), and treated with antarctic phosphatase (NEB) and polynucleotide kinase (NEB), according to the recommendations. We prepared strand-orientated libraries with the “Truseq Small RNA sample prep Kit” (Illumina). The final gel purification step has been replaced by a polymerase chain reaction cleanup with AMPureXP beads (Beckman-Coulter). We then proceeded to mRNA libraries illumina sequencing.

Small RNAs were extracted from total RNA of 50 pairs of ovaries and 100 pairs of testes, dissected from 2-to 4-day-old adults, using a TRIzol extraction according to the manufacturer’s procedure (TRIzol reagent, Invitrogen). We size fractionated small RNAs from 1 µg total RNA on a TBE-urea 15% acrylamide gel. We treated the resulting RNAs with the Illumina “Truseq Small RNA sample prep Kit” according to the manufacturer’s recommendations and send small RNA libraries to deep sequencing.

### Sequencing

Libraries were prepared and sequenced by the IMAGIF sequencing platform (Gif-sur-Yvette—France) on an Illumina Hiseq 1000 instrument, with a TruSeq SR Cluster Kit v3-cBot-HS (Illumina) and a TruSeq SBS v3-HS—50 cycles Kit (Illumina), using a single read 50-bp recipe. Libraries were pooled in equimolar proportions and diluted libraries to a final concentration of 12 pM, according to Illumina recommendations. The data were demultiplexed using the distribution of CASAVA software (CASAVA-1.8.2) (Mortazavi et al. 2008). The quality of the data was checked with the software FastQC 0.10.1 (available online at: http://www.bioinformatics.babraham.ac.uk/projects/fastqc).

### Read Mapping and Differential Expression Analysis

We filtered single-end reads from each library with UrQt software (Modolo and Lerat 2015); we retained only high quality reads (phred score > 33) for the analyses. We remove Illumina adapters using scythe software (https://github.com/najoshi/) and kept reads with a minimal length of 15 nucleotides. Quality-control data are presented in supplementary table S1, Supplementary Material online. We performed RNAseq mappings using the STAR software (Dobin et al. 2013) on the *Drosophila* TEs and reference genomes. We identified mRNA transcripts from TEs by mapping reads against a custom TE library derived from Repbase (Jurka et al. 2005). This database contains consensus sequences of all known TE in the *Drosophila* genomes. We kept reads mapping to a single consensus sequence (i.e., one TE family) and we generated count tables of TE transcripts with HTSeq. We performed differential expression analysis from these count matrices using the R bioconductor package DESeq2 (Love et al. 2014). The package DESeq2 implements a generalized linear model in which counts for each gene, in each sample, are modeled using a negative binomial distribution. We used one factor generalized linear model formulas depending on the tested conditions (i.e., species, population, or gonad). We selected differentially expressed TEs according to their adjusted *P* value (corrected *P* value < 0.1 and 10% false discovery rate). We compared DESeq2 results with another normalization method: TE expression was normalized by a pool of 100 housekeeping genes with stable expression between ovaries and testes samples (not shown). DESeq2 results were more stringent; we thus kept these in the main text. We used the same procedure to find differentially expressed genes mapping reads to the *D. melanogaster* and *D. simulans* reference genomes. Differential expression analysis of *pi*RNA pathway genes was performed on count matrices containing all orthologous genes shared by the two sibling species using DESeq2. Between versus within specific variations of TE transcription levels were compared using PCAs (implemented in DESeq2) on TE count matrices.

### Small RNA Mapping and Analysis

We removed barcodes and adapters from small RNA libraries of testes and ovaries using the Cutadapt tool and reads that are between 5 and 45 bp after stripping were kept. For each sample, we characterized small RNA species (supplementary table S1, Supplementary Material online). Then, we cleaned small RNA libraries from contaminant mRNA species (tRNA, rRNA, and genic mRNA in sense orientation). We identified TE-derived small RNA mapping small RNAs libraries (19– 30 nt) to our set of sex-biased TEs and nonsex-biased TEs by using bowtie (Langmead et al. 2009), allowing up to three mismatch and multiple matches to one position (-v [3] -M 1 –best –strata -p 12). The same analysis was performed with 0 mismatch (supplementary fig. S6, Supplementary Material online). All reads mapping to a unique TE consensus were pooled, and reads mapping to more than one TE consensus were discarded. To account for differences in sequencing depth between libraries and levels of sample contamination, the number of *pi*RNA per TE family was normalized by the total number of miRNAs, a *pi*RNA comigrant RNA species (supplementary table S3, Supplementary Material online). The ping-pong signature is the probability that a randomly sampled *pi*RNA from a given TE family has an antisense binding RNA overlapping on the first 10 bp. It was calculated using the tool described in Antoniewski (2014). In order to estimate *pi*RNA variation between populations and strains, we downloaded several sets of small RNA and compared them with our sequenced libraries (supplementary figs. S4 and S7, Supplementary Material online). To this end, we used two small RNA libraries presented supplementary table S1, Supplementary Material online.

### Comparison of Genome TE Content and Genome Annotation of TEs

We used the *Drosophila* Repbase data set (2,289 TE consensus) to identify TE insertions on recent releases of *D. melanogaster* and *D. simulans* reference genomes (dmel-r6.17 and dsim-r2.02) from which we removed contigs <15 kb (size of the longest TE in the data set).

From the Repbase TE list, we first discarded consensus sequences for which no RNAseq reads could map (449 sequences for *D. melanogaster* and 400 sequences for *D. simulans*) as queries for BlastN searches with default parameters (BlastN, *e*-value 10) on full genomes of both species. We removed all BLAST hits sharing <80% identity with TE consensus and merged successive hits belonging to the same TE family, when overlapping or when the lengths of the hits plus the gap distance in between were inferior to the size the TE consensus. When overlaps concerned different TE families, we kept the TE family with the highest identity to the consensus. In rare cases, some TE families displayed both overlapping and nonoverlapping regions. Insertions of this type were treated as two independent insertions. The final annotation files are summarized in supplementary table S2, Supplementary Material online. 

For each TE family, we aligned copies to their consensus using the Geneious mapper (high sensitivity; Geneious 10.2.3) and calculated the nucleotide diversity (*π*) using a custom script and the expression:

$$\pi =2\;.\sum_{i=1}^{n}\sum_{j=1}^{i-1}x_{i}x_{j}\pi_{ij},$$

where *x_i_* and *x_j_* are the respective frequencies of the *i*th and *j*th sequences, *π_ij_* is the number of nucleotide differences per nucleotide site between the *i*th and the *j*th sequences, and *n* is the number of sequences per TE family. All consensus genomic features with both normalized mRNA and *pi*RNA expression features are summarized supplementary table S3, Supplementary Material online, respectively, for *D. melanogaster* and *D. simulans*. Supplementary table S3, Supplementary Material online, also sum-up Pearson’ correlation statistics performed to highlight relationships between *pi*RNAs variations, TE genomic, and transcriptomic features.

### *pi*RNA Cluster Analysis

We extracted *pi*RNAs (23–30 nt) from our cleaned small RNA libraries and mapped these to both species reference genomes (dmel-r6.17 and dsim-r2.02). We mapped *pi*RNAs using bowtie, allowing up to one mismatch. We used a 1-kb window to identify all regions with densities greater than five *pi*RNA/kb. Only *pi*RNAs that uniquely mapped to the cluster were retained. Presence or absence of TEs was analyzed along each 1-kb window containing uniquely mapping *pi*RNAs. We obtained the total TE density along *pi*RNA cluster by averaging TE’ presence/absence by nucleotide position along 1-kb *pi*RNA cluster windows. We performed this analysis for each class TE according to their differential expression pattern. We summarized *pi*RNA cluster mapping for testes and ovaries in supplementary table S4, Supplementary Material online.

## Supplementary Material

evaa094_Supplementary_Data
