# Supplementary material for: piRNA and Transposon Dynamics in Drosophila: A Female Story
Source: Genome Biol Evol. 2020 May 12;12(6):931–47. doi: 10.1093/gbe/evaa094 (PMC7337185; doi:10.1093/gbe/evaa094)
Supplement: evaa094_Supplementary_Data [file evaa094_supplementary_data.zip › Figures(1).pdf]

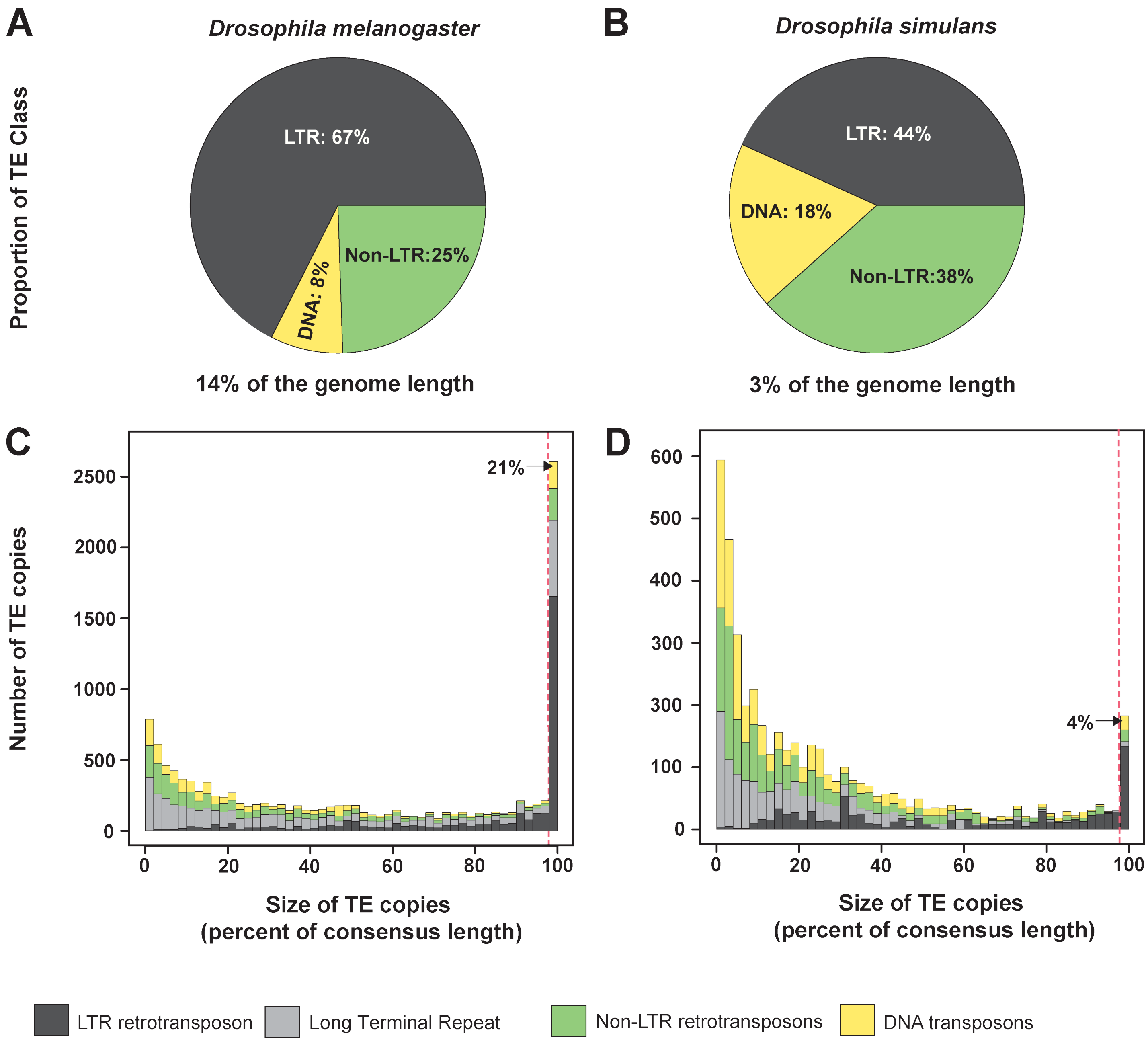

A
Drosophila melanogaster
Proportion of TE Class
LTR: 67%
Non-LTR:25%
DNA: 8%
14% of the genome length
B
Drosophila simulans
LTR: 44%
DNA: 18%
Non-LTR:38%
3% of the genome length
C
Number of TE copies
21%
Size of TE copies
(percent of consensus length)
D
4%
Size of TE copies
(percent of consensus length)
LTR retrotransposon
Long Terminal Repeat
Non-LTR retrotransposons
DNA transposons

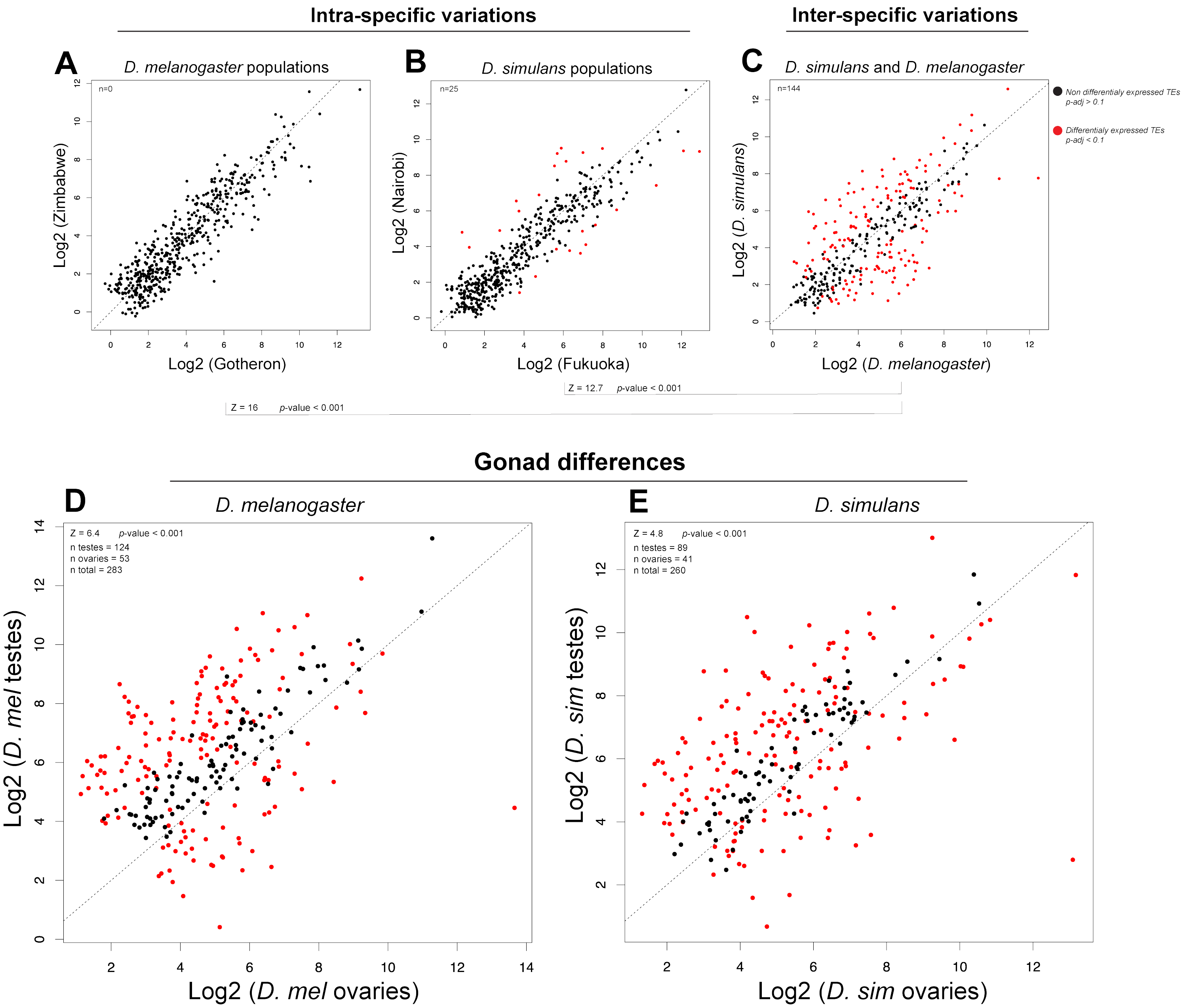
Intra-specific variations
Inter-specific variations
A
D. melanogaster populations
n=0
Log2 (Zimbabwe)
Log2 (Gotheron)
B
D. simulans populations
n=25
Log2 (Nairobi)
Log2 (Fukuoka)
C
D. simulans and D. melanogaster
n=144
Log2 (D. simulans)
Log2 (D. melanogaster)
Non differentialy expressed TEs p-adj > 0.1
Differentialy expressed TEs p-adj < 0.1
Z = 12.7 p-value < 0.001
Z = 16 p-value < 0.001
Gonad differences
D
D. melanogaster
Z = 6.4 p-value < 0.001
n testes = 124
n ovaries = 53
n total = 283
Log2 (D. mel testes)
Log2 (D. mel ovaries)
E
D. simulans
Z = 4.8 p-value < 0.001
n testes = 89
n ovaries = 41
n total = 260
Log2 (D. sim testes)
Log2 (D. sim ovaries)

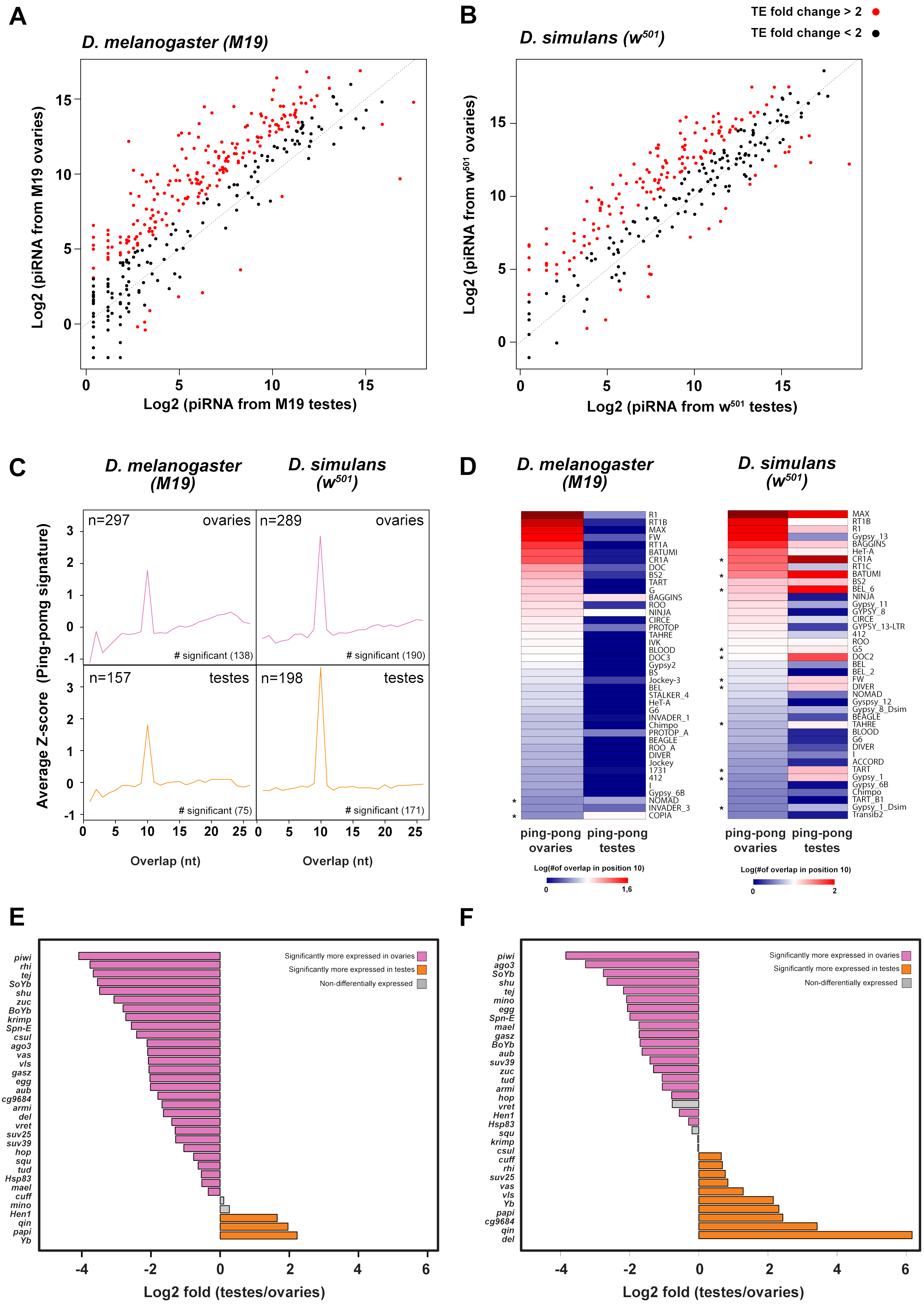
A
D. melanogaster (M19)
Log2 (piRNA from M19 ovaries)
Log2 (piRNA from M19 testes)
B
D. simulans (w501)
TE fold change > 2
TE fold change < 2
Log2 (piRNA from w501 ovaries)
Log2 (piRNA from w501 testes)
C
D. melanogaster (M19)
D. simulans (w501)
Average Z-score (Ping-pomg signature)
n=297
ovaries
# significant (138)
n=289
ovaries
# significant (190)
n=157
testes
# significant (75)
n=198
testes
significant (171)
Overlap (nt)
Overlap (nt)
D
D. melanogaster (M19)
R1
RT1B
MAX
FW
RT1A
BATUMI
CR1A
DOC
BS2
TART
G
BAGGINS
ROO
NINJA
CIRCE
PROTOP
TAHRE
IVK
BLOOD
DOC3
Gypsy2
BS
Jockey-3
BEL
STALKER_4
HeT-A
G6
INVADER_1
Chimpo
PROTOP_A
BEAGLE
ROO_A
DIVER
Jockey
1731
412
I
Gypsy_6B
NOMAD
INVADER_3
COPIA
ping-pong ovaries
ping-pong testes
Log(#of overlap in position 10)
0
1,6
D. simulans (w501)
MAX
RT1B
R1
Gypsy_13
BAGGINS
HeT-A
CR1A
RT1C
BATUMI
BS2
BEL_6
NINJA
Gypsy_11
GYPSY_8
CIRCE
GYPSY_13-LTR
412
ROO
G5
DOC2
BEL
BEL_2
FW
DIVER
NOMAD
Gypsy_12
Gypsy_8_Dsim
BEAGLE
TAHRE
BLOOD
G6
DIVER
I
ACCORD
TART
Gypsy_1
Gypsy_6B
Chimpo
TART_B1
Gypsy_1_Dsim
Transib2
ping-pong ovaries
ping-pong testes
Log(#of overlap in position 10)
0
2
E
piwi
rhi
tej
SoYb
shu
zuc
BoYb
krimp
Spn-E
csul
ago3
vas
vls
gasz
egg
aub
cg9684
armi
del
vret
suv25
suv39
hop
squ
tud
Hsp83
mael
cuff
mino
Hen1
qin
papi
Yb
Significantly more expressed in ovaries
Significantly more expressed in testes
Non-differentially expressed
Log2 fold (testes/ovaries)
F
piwi
ago3
SoYb
shu
tej
mino
egg
Spn-E
mael
gasz
BoYb
aub
suv39
zuc
tud
armi
hop
vret
Hen1
Hsp83
squ
krimp
csul
cuff
rhi
suv25
vas
vls
Yb
papi
cg9684
qin
del
Significantly more expressed in ovaries
Significantly more expressed in testes
Non-differentially expressed
Log2 fold (testes/ovaries)

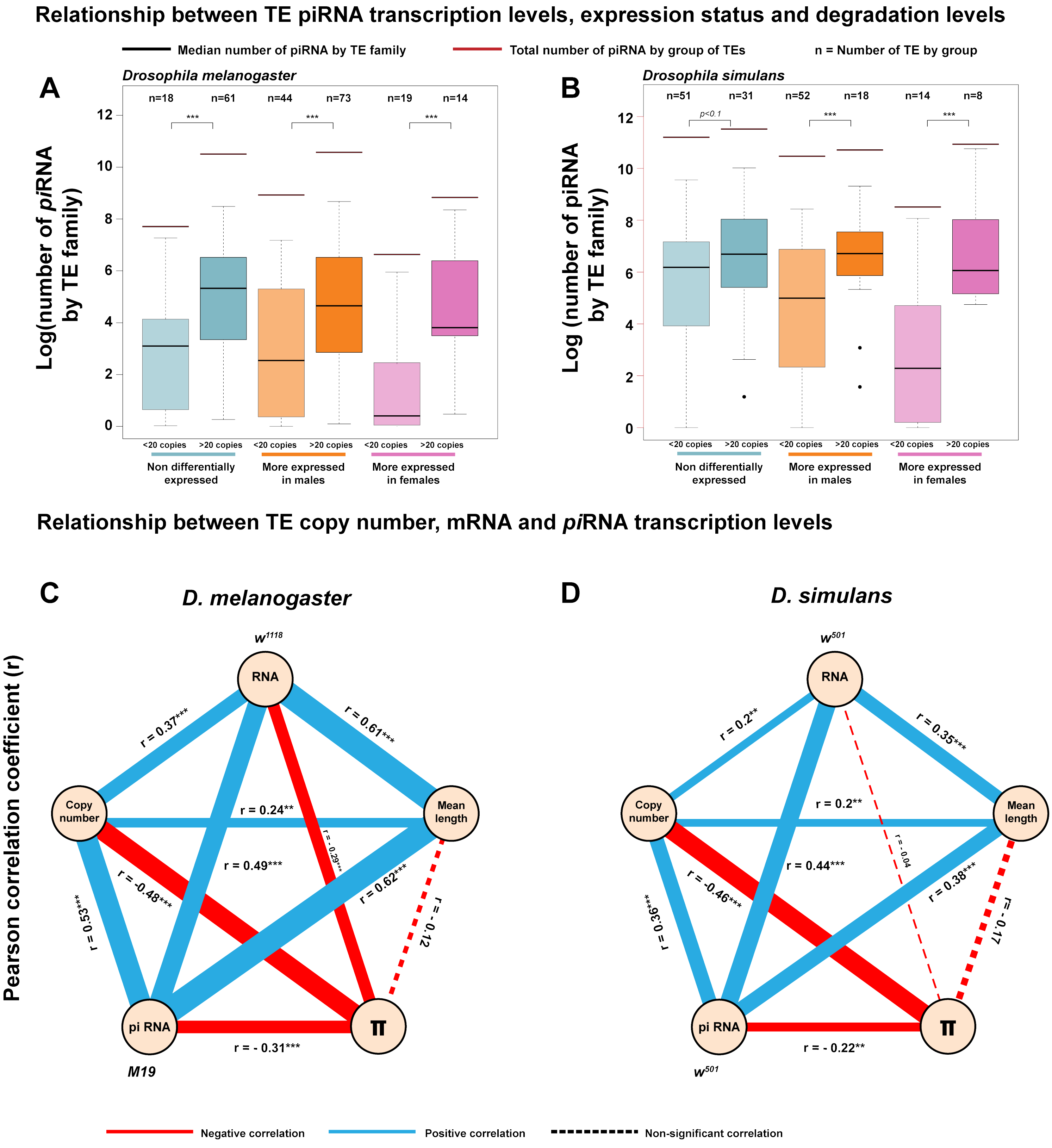
Relationship between TE piRNA transcription levels, expression status and degradation levels
Median number of piRNA by TE family
Total number of piRNA by group of TEs
n = Number of TE by group
A
Drosophila melanogaster
Log(number of piRNA by TE family)
n=18
n=61
n=44
n=73
n=19
n=14
***
<20 copies
>20 copies
Non differentially expressed
More expressed in males
More expressed in females
B
Drosophila simulans
Log (number of piRNA by TE family)
n=51
n=31
n=52
n=18
n=14
n=8
p<0.1
Relationship between TE copy number, mRNA and piRNA transcription levels
Pearson correlation coefficient (r)
C
D. melanogaster
w1118
RNA
Copy number
Mean length
pi RNA
π
M19
r = 0.37***
r = 0.61***
r = 0.24**
r = 0.49***
r = -0.29***
r = -0.48***
r = 0.62***
r = 0.53***
r = -0.12
r = - 0.31***
D
D. simulans
w501
r = 0.2**
r = 0.35***
r = 0.2**
r = 0.44***
r = -0.04
r = -0.46***
r = 0.38***
r = 0.36***
r = -0.17
r = - 0.22**
Negative correlation
Positive correlation
Non-significant correlation

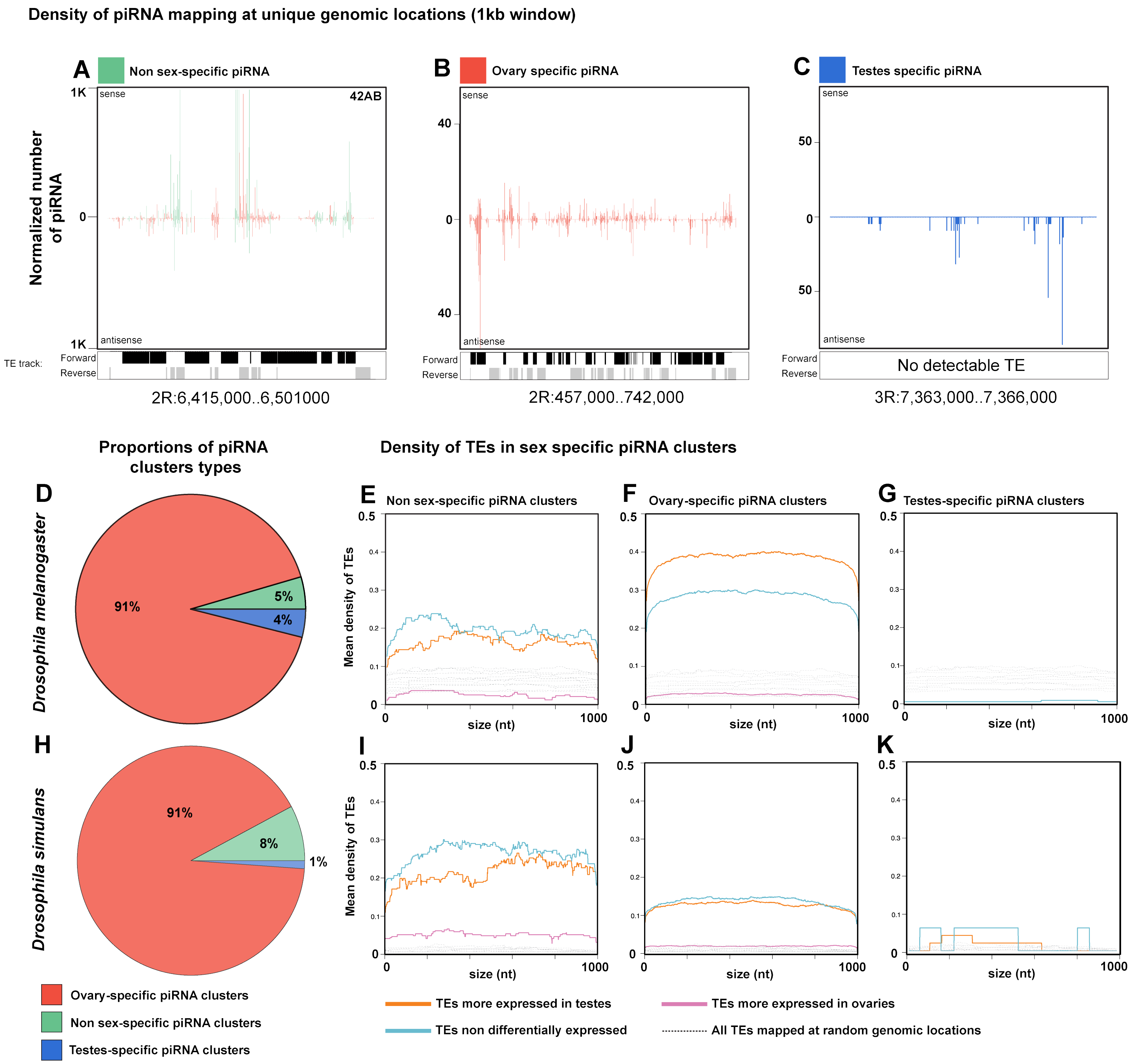

Density of piRNA mapping at unique genomic locations (1kb window)
A
Non sex-specific piRNA
B
Ovary specific piRNA
C
Testes specific piRNA
Normalized number of piRNA
sense
antisense
42AB
1K
0
40
50
TE track:
Forward
Reverse
No detectable TE
2R:6,415,000..6,501000
2R:457,000..742,000
3R:7,363,000..7,366,000
Proportions of piRNA clusters types
Density of TEs in sex specific piRNA clusters
D
Drosophila melanogaster
91%
5%
4%
E
Non sex-specific piRNA clusters
F
Ovary-specific piRNA clusters
G
Testes-specific piRNA clusters
Mean density of TEs
size (nt)
0
1000
0.5
H
Drosophila simulans
91%
8%
1%
I
J
K
Ovary-specific piRNA clusters
Non sex-specific piRNA clusters
Testes-specific piRNA clusters
TEs more expressed in testes
TEs non differentially expressed
TEs more expressed in ovaries
All TEs mapped at random genomic locations

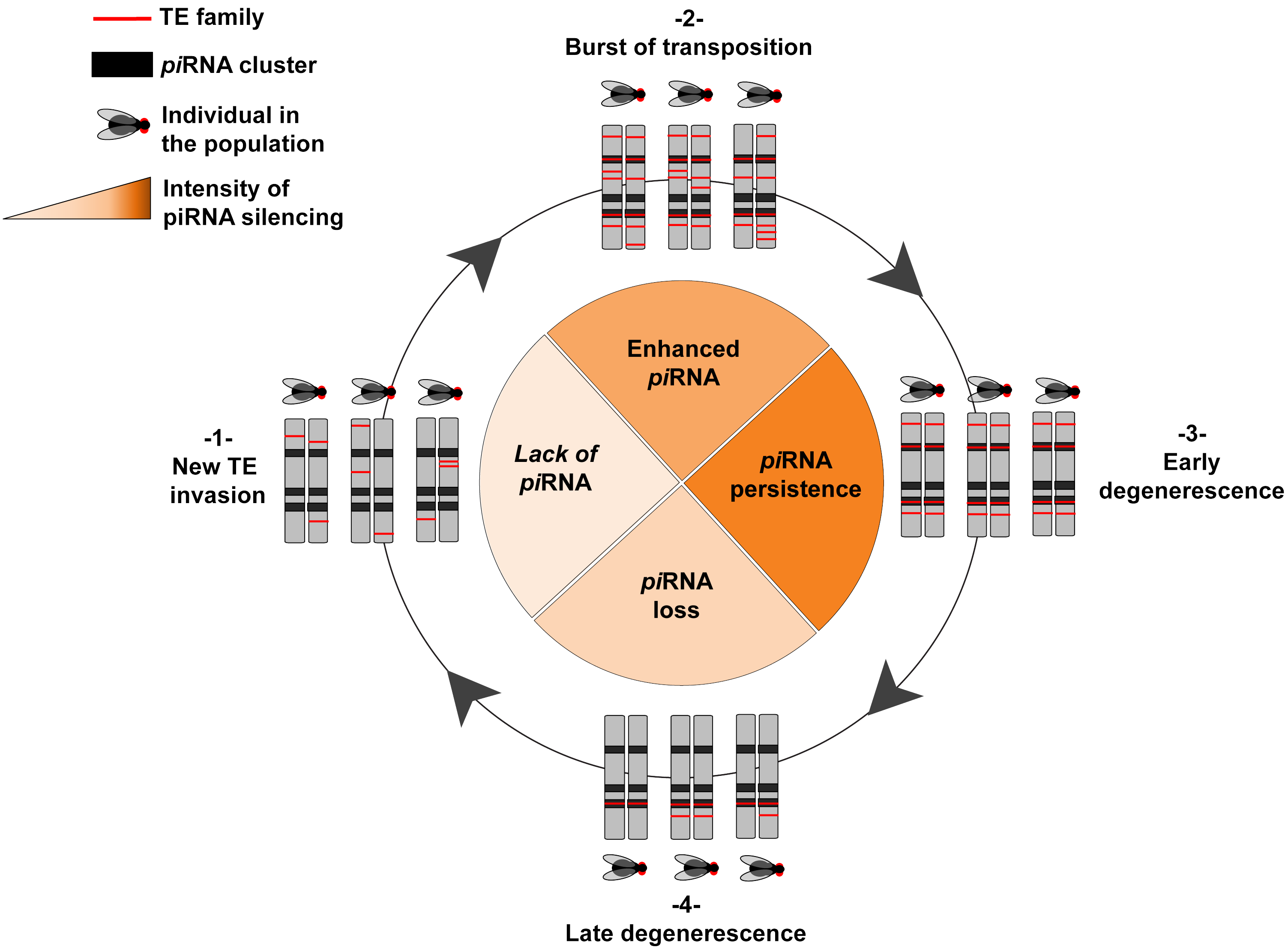

TE family
piRNA cluster
Individual in the population
Intensity of piRNA silencing
-2-
Burst of transposition
-1-
New TE invasion
-3-
Early degenerescence
-4-
Late degenerescence
Enhanced piRNA
Lack of piRNA
piRNA persistence
piRNA loss
