# Supplementary material for: piRNA and Transposon Dynamics in Drosophila: A Female Story
Source: Genome Biol Evol. 2020 May 12;12(6):931–47. doi: 10.1093/gbe/evaa094 (PMC7337185; doi:10.1093/gbe/evaa094)

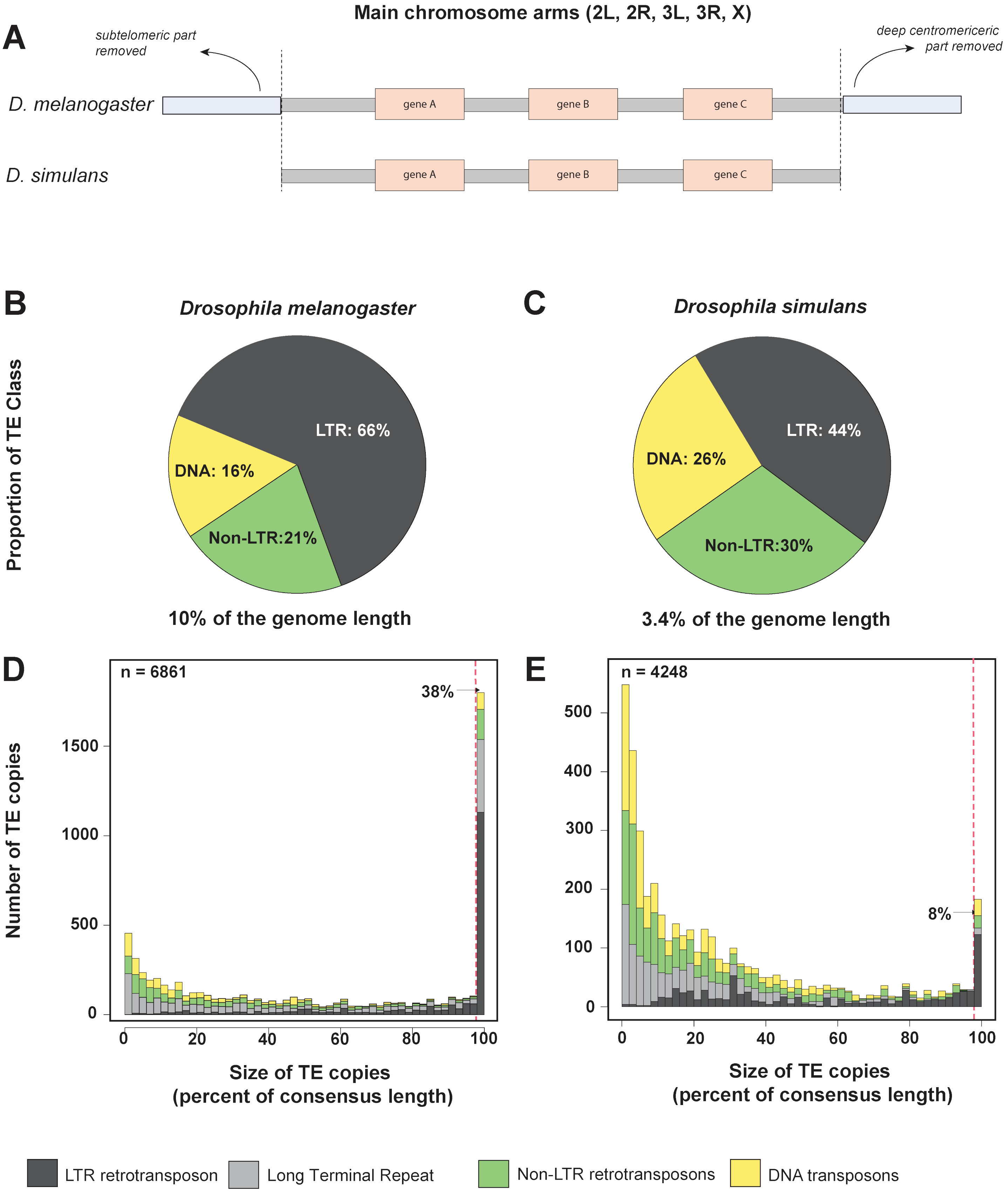
A
Main chromosome arms (2L, 2R, 3L, 3R, X)
subtelomeric part removed
deep centromericeric part removed
D. melanogaster
D. simulans
gene A
gene B
gene C
B
Drosophila melanogaster
Proportion of TE Class
LTR: 66%
DNA: 16%
Non-LTR:21%
10% of the genome length
C
Drosophila simulans
LTR: 44%
DNA: 26%
Non-LTR:30%
3.4% of the genome length
D
n = 6861
38%
Number of TE copies
Size of TE copies
(percent of consensus length)
E
n = 4248
8%
Size of TE copies
(percent of consensus length)
LTR retrotransposon
Long Terminal Repeat
Non-LTR retrotransposons
DNA transposons

PCA on TE levels of expression

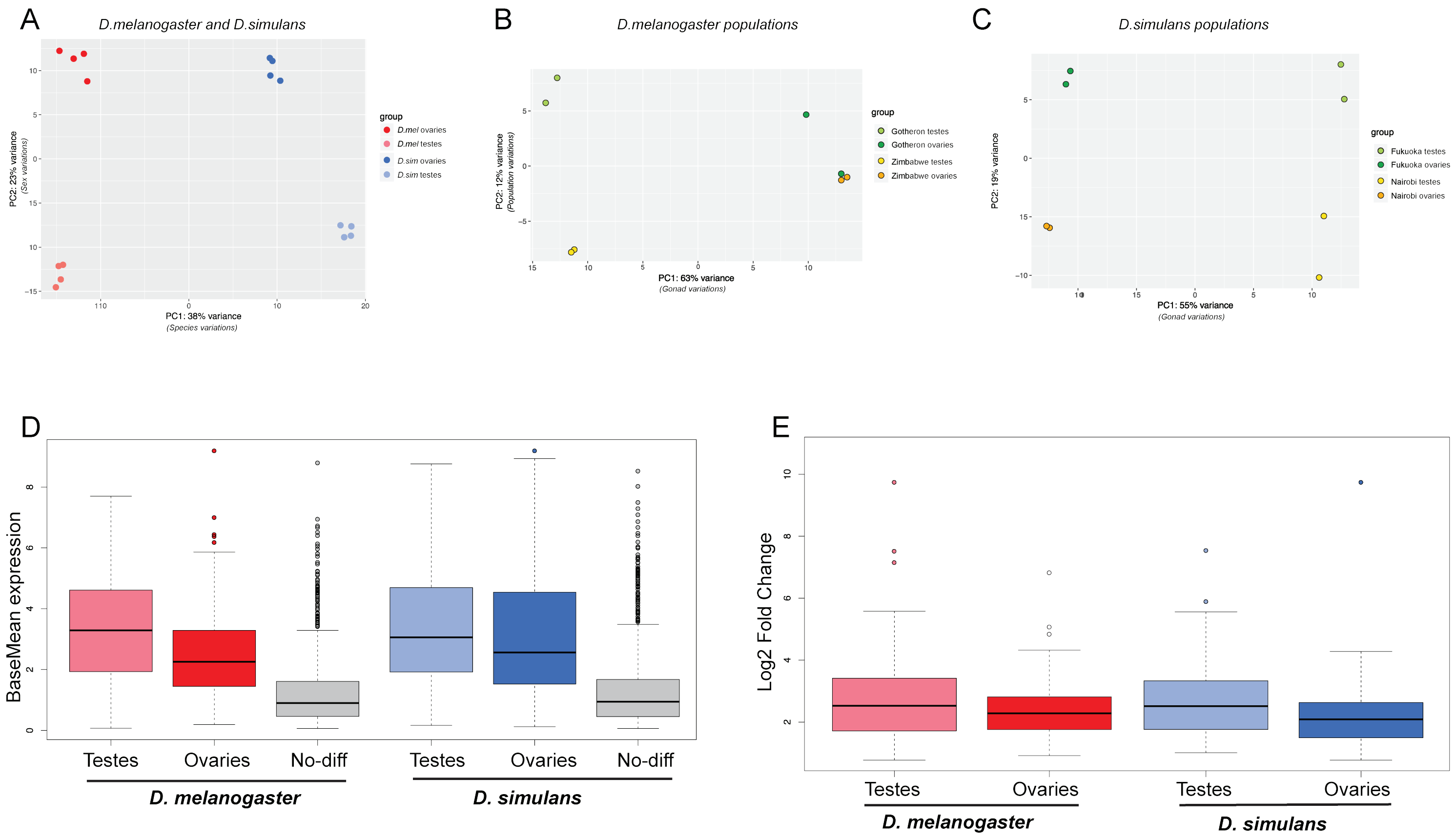

Proportions of differentially expressed TEs between testes and ovaries

A ***Drosophila melanogaster*** **B** ***Drosophila simulans***

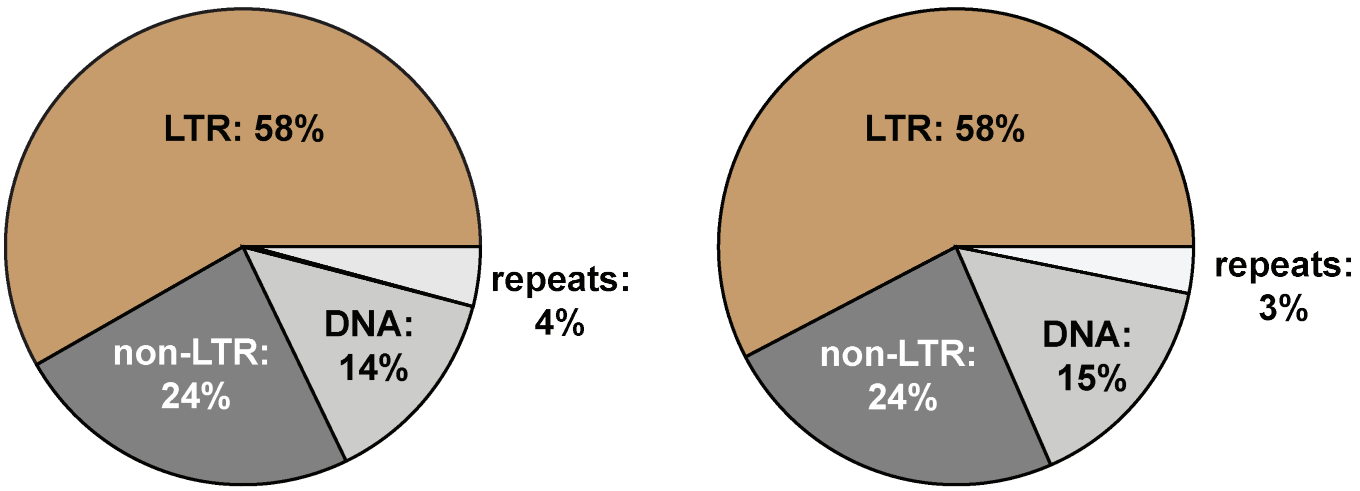

Differential expression analysis of TE-derived *pi*RNAs across populations, strains and tissues

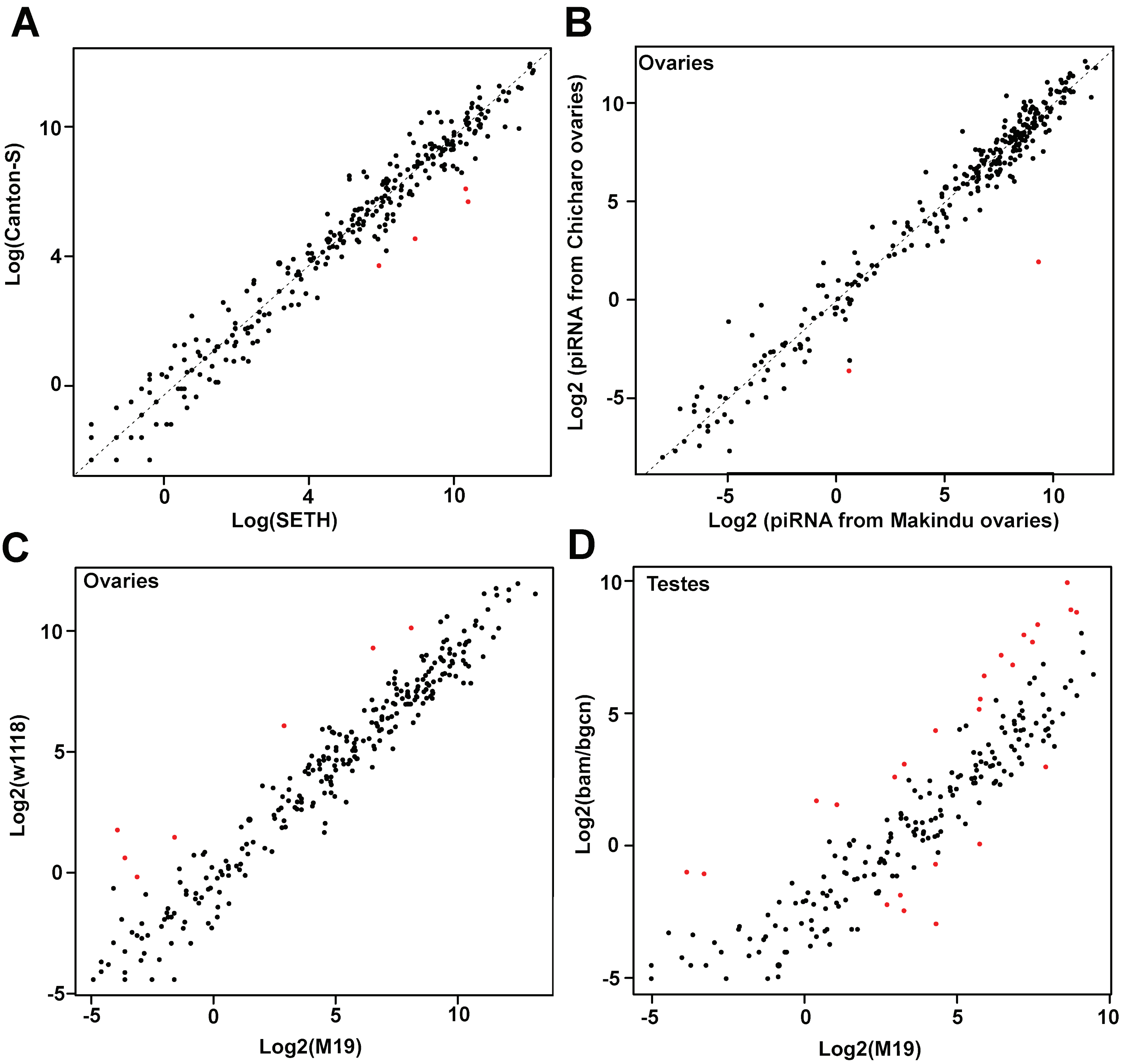

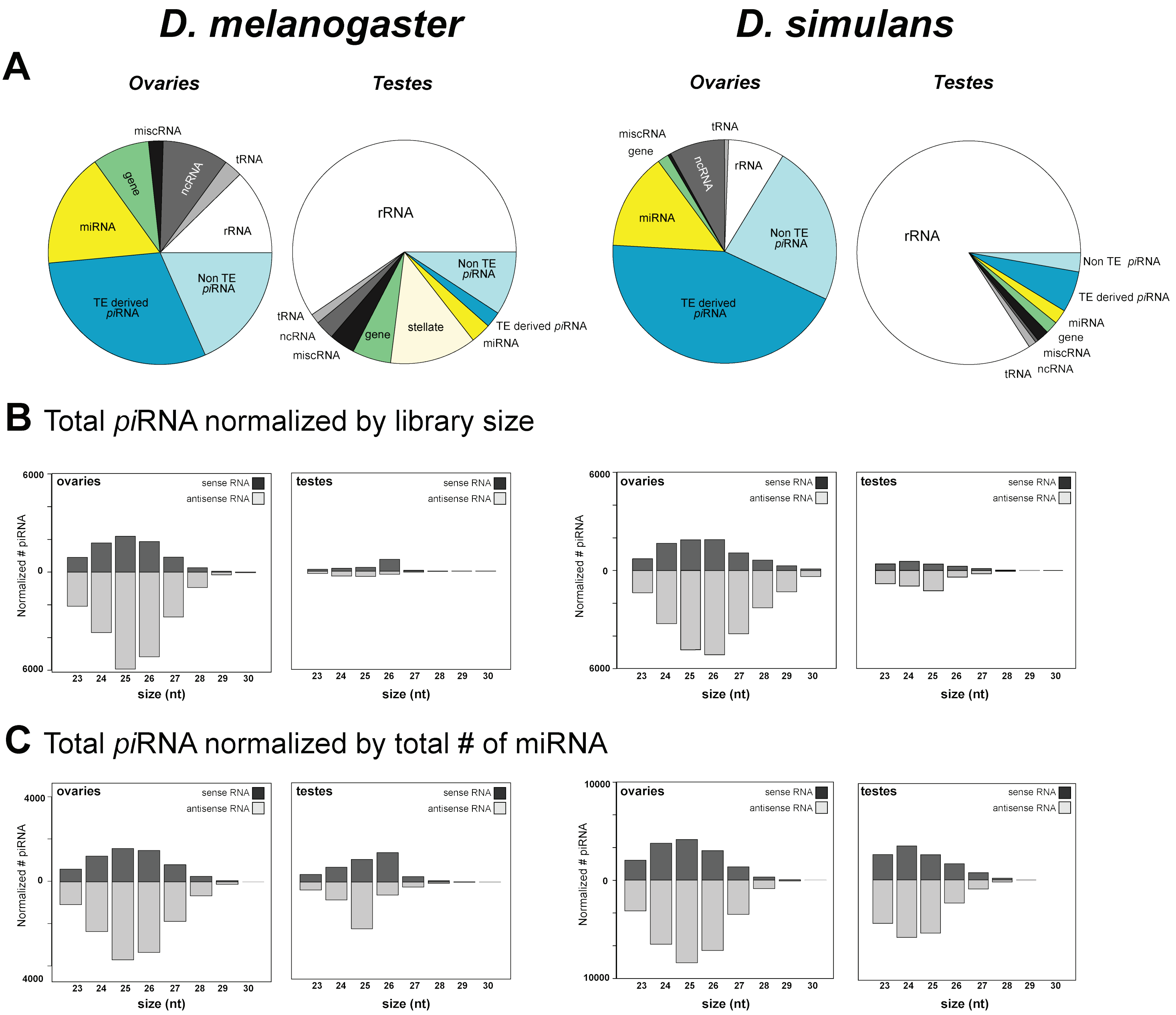

D. melanogaster
D. simulans
A
Ovaries
Testes
Ovaries
Testes
miscRNA
tRNA
gene
ncRNA
miRNA
rRNA
Non TE piRNA
TE derived piRNA
stellate
B Total piRNA normalized by library size
ovaries
testes
sense RNA
antisense RNA
Normalized # piRNA
size (nt)
C Total piRNA normalized by total # of miRNA

Differentialy expressed TE-derived *pi*RNAs between gonads (mapped with 0 mismatch)

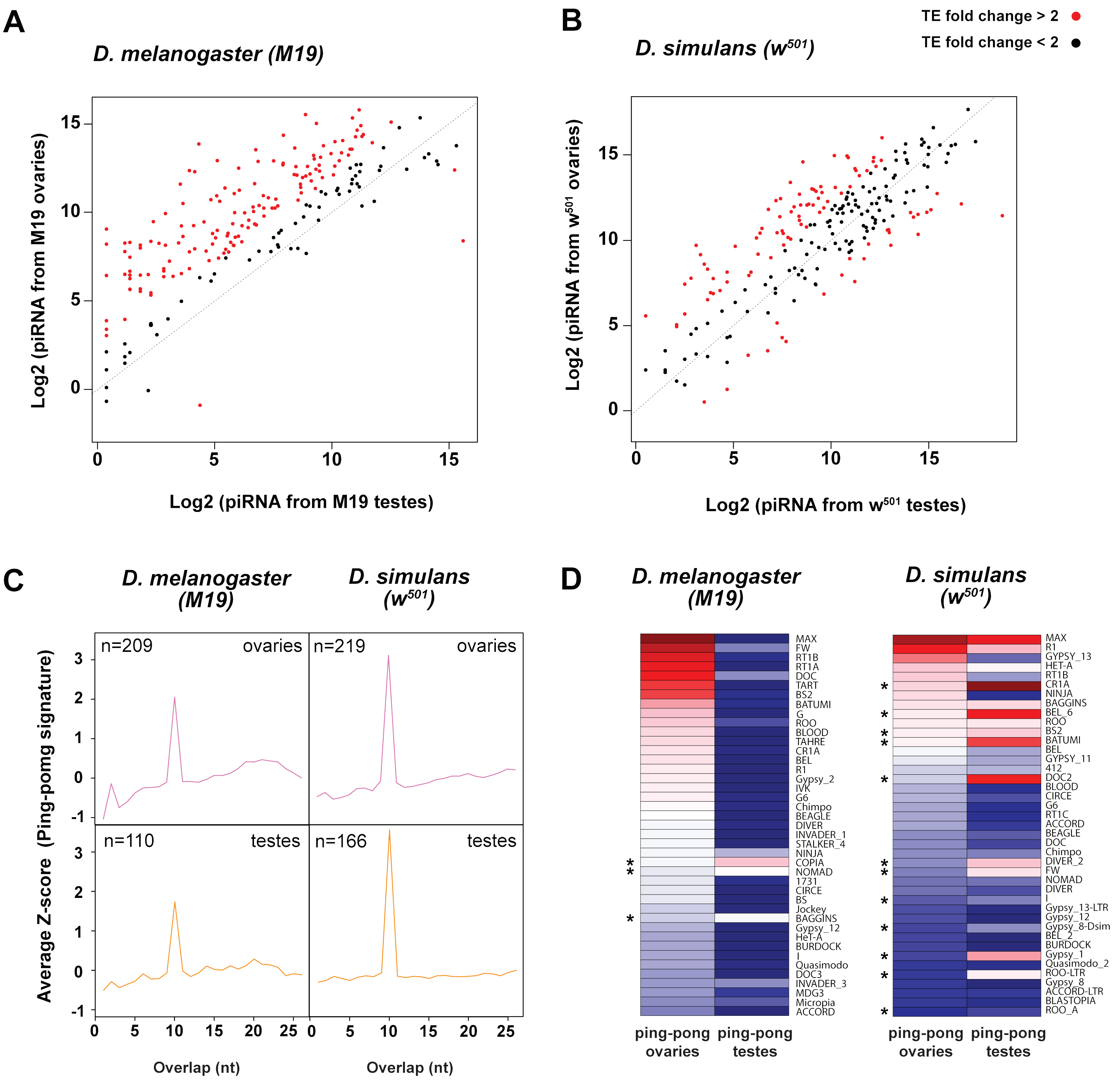

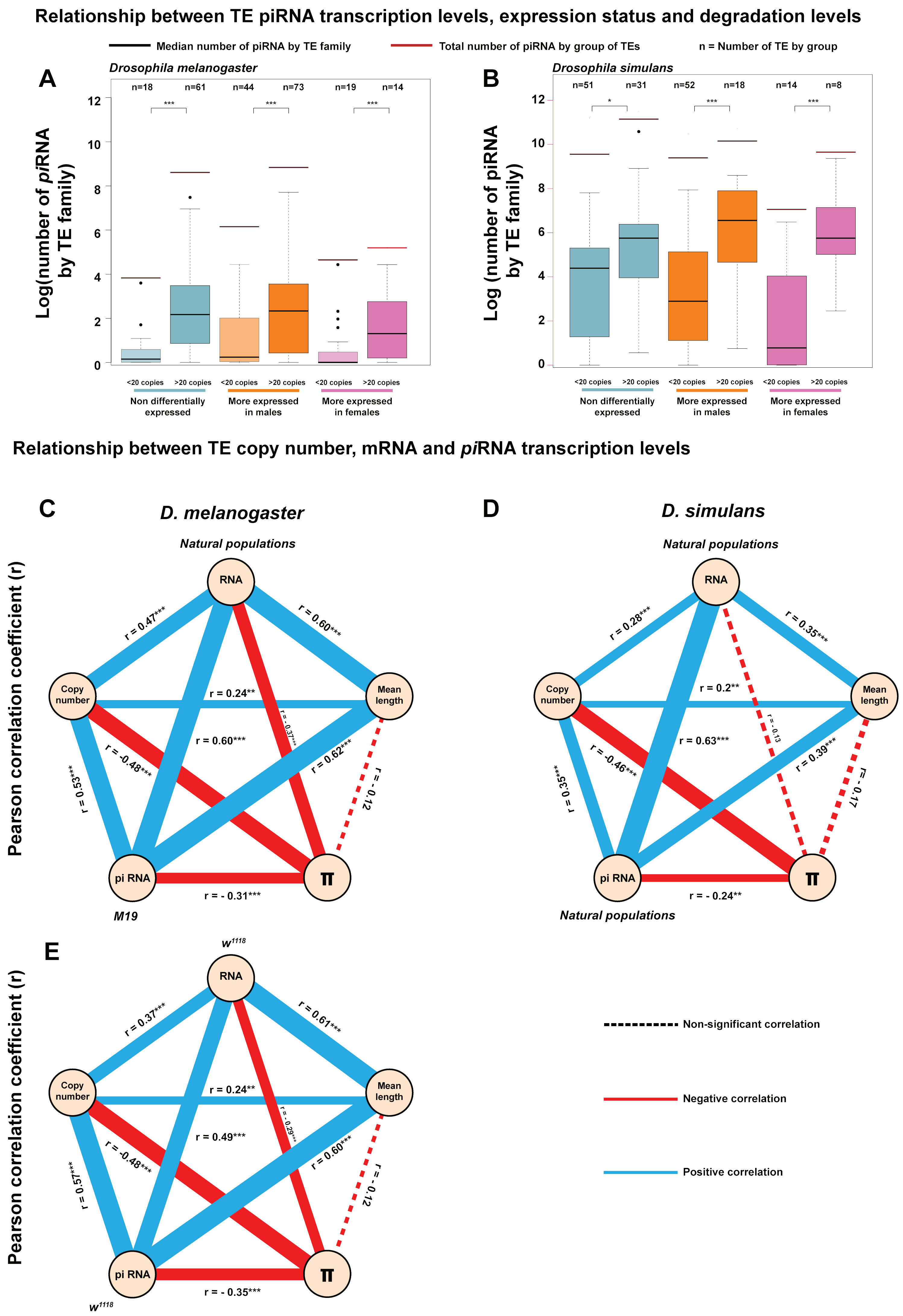
Relationship between TE piRNA transcription levels, expression status and degradation levels
Median number of piRNA by TE family
Total number of piRNA by group of TEs
n = Number of TE by group
A
Drosophila melanogaster
Log(number of piRNA by TE family)
n=18
n=61
n=44
n=73
n=19
n=14
***
***
***
<20 copies
>20 copies
Non differentially expressed
More expressed in males
More expressed in females
B
Drosophila simulans
Log (number of piRNA by TE family)
n=51
n=31
n=52
n=18
n=14
n=8
*
***
***
Relationship between TE copy number, mRNA and piRNA transcription levels
C
D. melanogaster
Natural populations
Pearson correlation coefficient (r)
RNA
Copy number
Mean length
pi RNA
π
r = 0.47***
r = 0.60***
r = 0.24**
r = 0.60***
r = -0.37***
r = -0.48***
r = 0.62***
r = 0.53***
r = -0.12
r = - 0.31***
M19
D
D. simulans
Natural populations
r = 0.28***
r = 0.35***
r = 0.2**
r = 0.63***
r = -0.13
r = -0.46***
r = 0.39***
r = 0.35***
r = -0.17
r = - 0.24**
Natural populations
E
w1118
r = 0.37***
r = 0.61***
r = 0.24**
r = 0.49***
r = -0.29***
r = -0.48***
r = 0.60***
r = 0.57***
r = -0.12
r = - 0.35***
w1118
Non-significant correlation
Negative correlation
Positive correlation

*pi*RNA orientation (sense or antisense) between sex-specific cluster

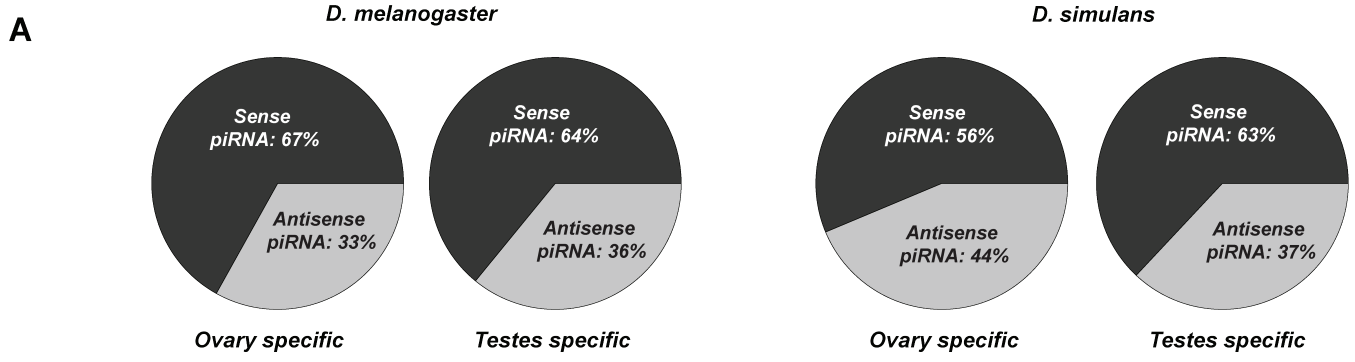

Density of TEs within sex-specific piRNA clusters (comparison of both species after the removal of deep heterochromatin regions)

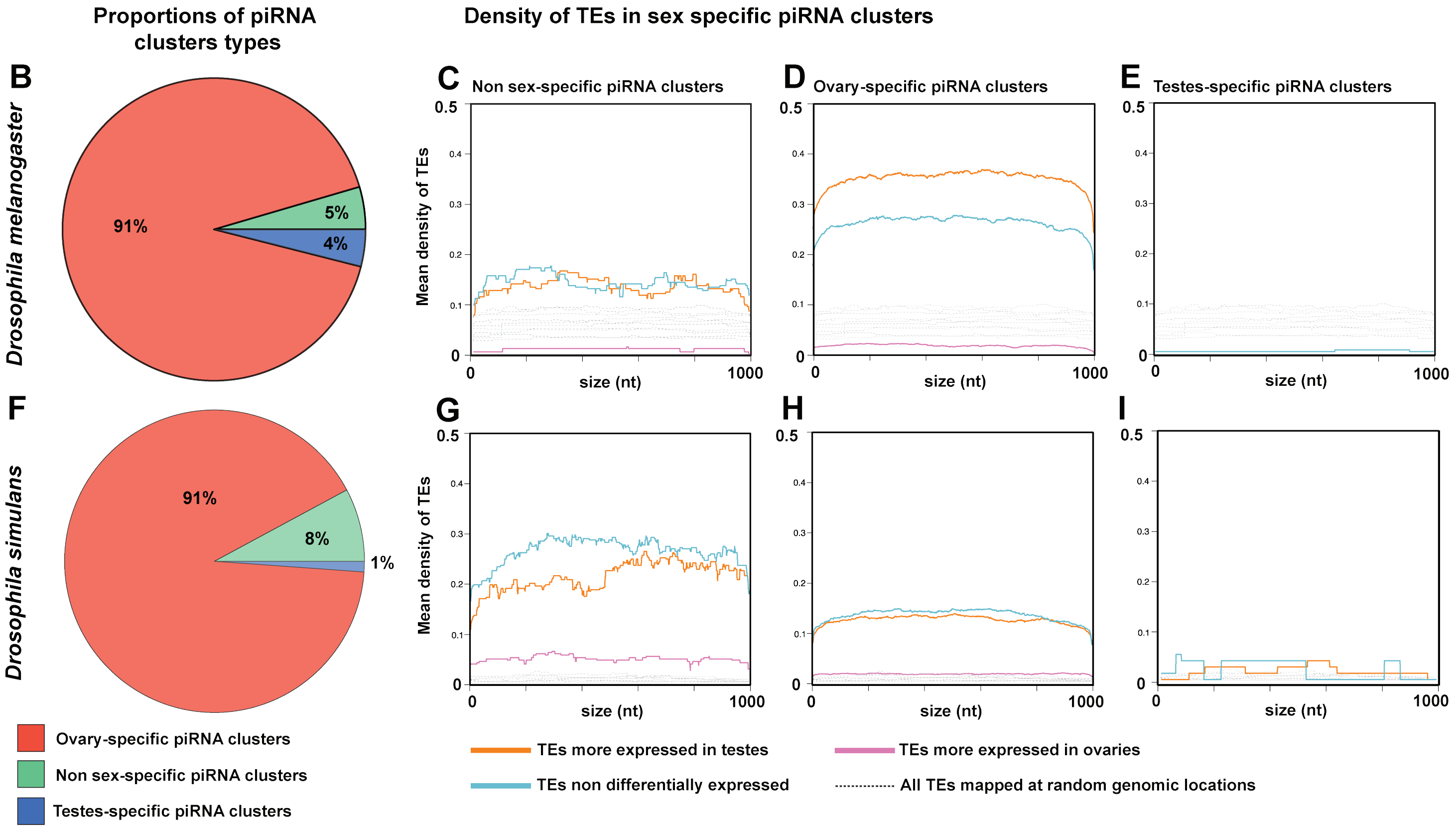

Supplement: evaa094_Supplementary_Data [file evaa094_supplementary_data.zip › Supplementary(1).pdf]
